# The Clinical Validity and Utility of PCR Compared to Conventional Culture and Sensitivity Testing for the Management of Complicated Urinary Tract Infections in Adults: A Secondary (Ad Hoc) Analysis of Pathogen Detection, Resistance Profiles, and Impact on Clinical Outcomes

**DOI:** 10.3390/microorganisms13040949

**Published:** 2025-04-20

**Authors:** Moustafa Kardjadj, Tara W. Chang, Roel Chavez, DeAndre Derrick, Frank L. Spangler, Itoe P. Priestly, Lauren Y. Park, Thomas K. Huard

**Affiliations:** 1 dicentra, Toronto, ON M4W 3E2, Canada; 2 Albany Urology Clinic & Surgery Center, Albany, GA 31707, USA; 3 Doc Lab Inc., Hillsboro, OR 97006, USA; 4 Soft Cell Laboratories, St. George, UT 84770, USA; 5 MED-US Consulting, LLC., Austin, TX 78734, USA

**Keywords:** urinary tract infection, polymerase chain reaction, microbial sensitivity tests, antimicrobial drug resistance, polymicrobial infections, molecular diagnostic testing, patient-relevant outcome

## Abstract

Clinical success in treating complicated urinary tract infections (cUTIs) depends on accurate pathogen detection, given the common occurrence of polymicrobial infections and antimicrobial resistance. This multicenter, randomized, investigator-blinded study compared polymerase chain reaction (PCR)-based diagnostics to conventional culture and sensitivity (C&S) testing in guiding the treatment of cUTIs. PCR identified polymicrobial infections in 43.52% of cases, a significantly higher rate than that observed with C&S (31.95%, *p* = 0.033). Patients in the C&S arms with undetected polymicrobial infections had a significantly higher clinical failure rate (33.33%, 14/42, *p* = 0.041) compared to those with concordant polymicrobial infection identification by both methods (22.22%, 12/54). PCR also detected additional pathogens in 54.44% (92/169) of cases in the C&S arm, where clinical failure was significantly higher when C&S missed pathogens (28.26% vs. 14.29%, *p* = 0.015). Similarly, when C&S failed to detect phenotypic resistance (compared to PCR), clinical failure occurred in 50% (16/42) of cases, compared to 13.22% (21/121, *p* = 0.001) when resistance detection was concordant (PCR and C&S). To further illustrate the clinical impact, patient-level case analyses are included to demonstrate how PCR-guided therapy improved pathogen detection and enabled more appropriate antimicrobial selection compared to C&S. These findings highlight the limitations of C&S in detecting polymicrobial infections, antimicrobial resistance, and hetero-resistance due to its limited clonal analysis, supporting the integration of PCR for more accurate diagnostics and optimized cUTI management.

## 1. Introduction

Complicated urinary tract infections (cUTIs) present a significant clinical challenge due to their complex microbial etiology, rising antimicrobial resistance, and potential for severe morbidity and mortality [1]. Rapid and accurate pathogen identification is crucial for optimizing antimicrobial therapy and improving patient outcomes. While conventional culture and sensitivity (C&S) testing is the standard diagnostic approach, its prolonged turnaround time can delay effective treatment initiation. In contrast, polymerase chain reaction (PCR)-based diagnostics enable the rapid detection of uropathogens, including the identification of resistance genes, which has the potential to influence early therapeutic decisions and enhance antimicrobial stewardship [2,3].

A randomized controlled trial evaluated the clinical validity and utility of PCR versus C&S for managing adult cUTIs [4,5]. A total of 773 patients were enrolled in the study. Both diagnostic tests were performed for all participants before the randomization assignment; however, investigators remained blinded to the comparator test results throughout the trial. To ensure unbiased treatment decisions, clinicians were blinded to the comparator test results. Treatment was prescribed solely based on the assigned test—PCR results for the PCR arm and C&S results for the C&S arm. The comparator test results remained blinded until the end of the study (EOS) [4,5].

The robust study design enabled the collection of a comprehensive dataset, with both PCR and C&S results for all participants at baseline and end of study (EOS), irrespective of their assigned group. Additionally, the dataset includes gene resistance markers detected by PCR and phenotypic sensitivity profiles from C&S, allowing for a direct comparison of antimicrobial resistance patterns. Data on recommended antibiotics—based on genotypic (PCR) and phenotypic (C&S) resistance profiles—as well as prescribed antibiotics—based on the assigned diagnostic result—were also captured. Clinical outcomes were systematically categorized as clinical failure (CF) or favorable clinical outcome (FCl), enabling a detailed assessment of treatment efficacy. This extensive dataset provides a unique opportunity for a direct comparison of the diagnostic accuracy, therapeutic impact, and clinical outcomes of both testing methods. Unlike studies that evaluate either PCR or C&S in isolation, this trial’s dual-test approach allows for a comprehensive, side-by-side evaluation of their relative performance, offering valuable insights into their clinical utility [4,5].

The first two parts of this study yielded critical insights into the diagnostic performance and clinical impact of PCR versus C&S in managing cUTIs. Part I demonstrated that PCR testing significantly reduced diagnostic turnaround time, with a mean of 49.68 h in the PCR arm compared to 104.4 h in the C&S arm (*p* < 0.001). This reduction facilitated faster treatment modifications and led to higher clinician satisfaction scores (23.95 ± 1.96 vs. 20.64 ± 4.12, *p* < 0.001). Additionally, the FCl rates were significantly higher in the PCR arm (88.08%) compared to the C&S arm (78.11%, *p* = 0.011), particularly among patients who have high risk factors (female, older, and with polymicrobial infection) [4].

Part II further highlighted that PCR-guided therapy influenced antimicrobial prescribing patterns, favoring the use of oral antibiotics, which achieved an 87.15% FCl, significantly higher than the 77.37% FCl observed in the C&S group (*p* = 0.018). The study also confirmed strong agreement between PCR and C&S in diagnosing positive cases (88.06% at EOS) but revealed notable discordance in negative cases (62.91%), primarily due to C&S limitations in detecting uropathogens. PCR detected and guided treatment for infections missed by C&S, leading to better clinical outcomes. Additionally, PCR identified a broader range of pathogens, including polymicrobial infections often overlooked by C&S [4,5]. These findings emphasize the potential clinical advantages of PCR in improving diagnostic accuracy, guiding antimicrobial selection, and enhancing patient outcomes [5].

This Part III paper presents an ad hoc evaluation of the full trial dataset. The analyses reported here were not part of the pre-specified clinical utility endpoints and have not been previously published in Parts I or II [4,5]. This analysis focuses on the impact of PCR on pathogen detection, polymicrobial case identification, antibiotic resistance profiling, treatment decisions, and clinical outcomes. By leveraging the full dataset, this study provides critical insights into the diagnostic and therapeutic implications of PCR-based testing for cUTI management. Patient-level case analyses further illustrate the clinical impact, demonstrating how PCR-guided therapy influenced treatment decisions and improved outcomes compared to C&S-based management. These case-based evaluations offer a more granular perspective on the real-world application of PCR in optimizing antimicrobial therapy and reducing CFs.

## 2. Materials and Methods

### 2.1. Study Design

**Figure 1:**
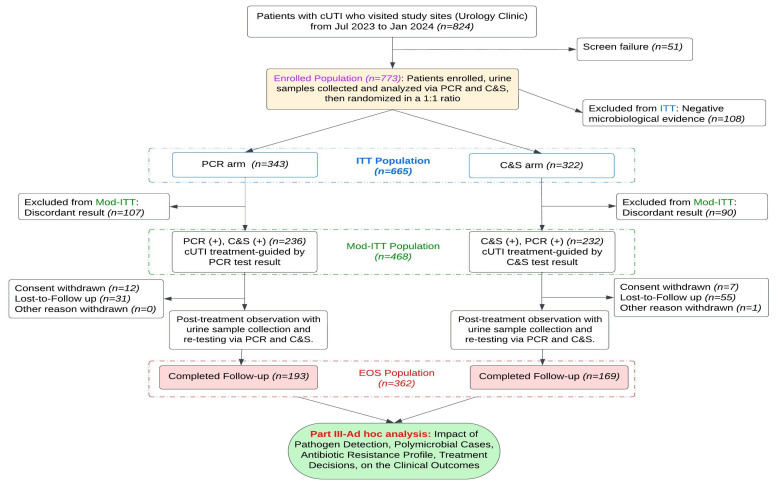
The CONSORT flow diagram, detailing the schedule of events throughout the study. Both the PCR and C&S testing were performed for all enrolled participants (*n* = 773) before randomization into one of the two study arms. Investigators made clinical decisions exclusively based on the diagnostic results from their assigned arm, remaining blinded to the comparator test results until EOS. As per study protocol requirements, certain cases were excluded from specific analyses, including 108 patients with negative microbiological evidence by both diagnostic methods, 197 discordant cases (90 from the C&S arm and 107 from the PCR arm), and 108 patients who did not complete all study procedures (19 withdrew consent, 86 were lost to follow-up, and 1 was excluded for other reasons). These cases were reported in detail in Part I and Part II of the study [4,5].

The study was designed as a multi-center, randomized, parallel, investigator-blind clinical trial conducted across six sites with regional variance to evaluate the clinical utility of molecular testing by comparing the diagnostic and therapeutic outcomes of PCR-based molecular diagnostics with conventional urine C&S in managing cUTIs in adults. The overall study design is outlined in Figure 1. Eligible patients who met all inclusion criteria and none of the exclusion criteria provided informed consent before enrollment. Urine samples were collected from all participants and tested using both PCR and C&S methods before randomization. Participants were randomized in a 1:1 ratio to receive treatment guided by either the PCR or C&S results. However, clinician investigators were blinded to the comparator test results and prescribed treatment based solely on the assigned diagnostic modality. Patients in the PCR arm received therapy based exclusively on PCR results, while those in the C&S arm were treated according to standard C&S findings.

The study was approved by an independent Institutional Review Board (Advarra IRB ID: Pro00071764 Date: 22 May 2023) and its conduct was overseen by an independent contract research organization (dicentra CRO, Study ID: 22-UPHUV−01) to ensure regulatory compliance and minimize potential bias.

### 2.2. Laboratory Testing Procedures

Urine specimens were collected using a clean-catch midstream technique at two distinct time points—at baseline and post-treatment reassessment—following targeted therapy based on the assigned diagnostic arm. Collected samples were immediately stored at 2–8 °C until analysis to preserve sample integrity. Each specimen was labeled with the subject identification, method of collection, date, and time of collection to ensure proper traceability.

Upon receipt, urine samples were aliquoted for parallel testing, with one portion analyzed using PCR (QuantStudio 7 or 12 and KingFisher) and the other processed using C&S methodologies (Supplementary Materials). The C&S aliquots underwent culturing and quantification using a calibrated loop, with a bacterial growth threshold set at ≥10⁵ CFU/mL for positivity. Isolated pathogens were subjected to species identification and antimicrobial sensitivity testing.

Molecular testing (Doc Lab UTM 2.0) involved qualitative PCR amplification targeting 28 uropathogen species and 16 classes of antibiotic resistance genes, covering both bacterial and fungal pathogens. This approach enabled rapid pathogen identification and early detection of resistance markers, offering a comprehensive assessment of the microbial and resistance landscape in cUTI cases. Doc Lab UTM 2.0 ‘s analytical performance characteristics were reported in Part II [5]

All urine samples for urine C&S were shipped to the central laboratory. The urine culture, isolation of uropathogen(s), initial identification of pathogen(s), and bacterial counts in urine were conducted in the central laboratory. The urine samples were cultured and quantified using a calibrated loop to identify a quantitative count of bacteria at a lower limit of 10^5^ CFU/mL. All purified pathogen(s) were further analyzed for species identification and antimicrobial sensitivity.

### 2.3. Interpretation of Resistance Markers and Treatment Selection

The genetic resistance markers detected via PCR were considered critical in determining antibiotic selection. The interpretation of these markers was guided by Clinical and Laboratory Standards Institute (CLSI) guidelines, with clinicians allowed discretion in assessing their clinical significance. Resistance markers known to strongly correlate with phenotypic resistance, such as *blaKPC* (carbapenem resistance) and *mecA* (methicillin resistance), were treated as absolute indicators of resistance. Other markers with variable phenotypic expression, such as *qnr* genes (fluoroquinolone resistance), were interpreted with clinical judgment.

### 2.4. Treatment Decisions and Polymicrobial Considerations

All detected organisms in the PCR arm were treated equally, leading to the frequent use of appropriate antibiotics. The higher rate of polymicrobial detection in the PCR group influenced antibiotic selection, favoring agents with appropriate coverage. Clinicians selected antibiotic regimens independently, based solely on their assigned test results, without access to the comparator’s findings.

### 2.5. Study and Analysis Population

This ad hoc analysis included two distinct study populations:

Enrolled Population: This group comprised all enrolled and randomized patients with available PCR and C&S diagnostic results. This population was used only to assess PCR performance at the start of the study, specifically, its sensitivity and specificity, stratified by clinical and demographic characteristics.

EOS Population: This group included all enrolled patients who completed the study procedures, including the treating investigators’ evaluation. This population was used to evaluate PCR performance in terms of sensitivity and specificity at EOS and to analyze the impact of PCR on pathogen detection, polymicrobial case identification, antibiotic resistance profiles, treatment decisions, and clinical outcomes.

Clinical treatment outcomes were assessed by the clinician at study completion. **Favorable clinical outcomes (FCl)** were defined as the investigator’s questionnaire-based assessment indicating the resolution of at least one baseline cUTI symptom, the absence of new symptoms, and/or the avoidance of parenteral antibiotic therapy following randomization. **Clinical failure (CF)** was defined as the presence of persistent or new cUTI symptoms, as reported in the EOS assessment.

### 2.6. Statistical Analysis

Descriptive and trend analyses were conducted for all variables. Continuous variables were reported as means ± standard deviation (SD) or ranges, as appropriate, while categorical variables were expressed as percentages. Statistical analyses were performed using R software -Core Team 2024 (R−4.4.2, Company, Vienna, Austria) and RStudio Team 2024 (RStudio−3.6.0 +, Boston, MA, USA) for Windows.

All statistical tests were two-sided, and a *p*-value < 0.05 was considered statistically significant. Categorical variables were analyzed using the Chi-square test or Fisher’s exact test when expected frequencies were <5, while continuous variables were compared using the Student’s t-test or Mann–Whitney U test for non-normally distributed data.

Relative risk (RR) with 95% confidence intervals (CI) was calculated to assess the likelihood of clinical outcomes in different groups. RR was determined as the ratio of the probability of clinical outcome in the exposed group to the probability of clinical outcome in the reference group. Confidence intervals for RR were computed using the Wald method, and statistical significance was assessed using the z-test for RR estimates.

## 3. Results

### 3.1. Test Performance Across Clinical and Demographic Subpopulations

We evaluated (Table 1) the agreement between PCR and conventional C&S results in both the enrolled population (*n* = 773) and the end-of-study (EOS) population (*n* = 362). In the enrolled population, PCR demonstrated high sensitivity (95.32%; defined as the proportion of C&S-positive cases detected by PCR) but lower specificity (38.30%; defined as the proportion of C&S-negative cases correctly identified by PCR). In the EOS population, the sensitivity was 88.06% and the specificity was improved to 62.91%. Subgroup analyses across study sites, age, sex, symptom burden, and infection type revealed consistent trends. For example, in the EOS population, elderly patients (≥65 years) exhibited a sensitivity of 85.12% and a specificity of 62.45%, while non-elderly patients (<65 years) showed a sensitivity of 88.74% with a specificity of 63.88%.

### 3.2. Polymicrobial Detection and Impact on Clinical Outcomes

The impact of polymicrobial versus monomicrobial infections on clinical outcomes was assessed within each diagnostic arm (PCR vs. C&S). Table 2 summarizes the distribution of monomicrobial and polymicrobial infections detected in each arm, along with their corresponding clinical outcomes.

To evaluate the clinical consequences of undetected polymicrobial infections, an analysis of cases where PCR-detected polymicrobial infections were missed by C&S (Table 2 and Table 3) was performed. Among the 42 patients in the C&S arm whose C&S results failed to detect polymicrobial infections, the CF rate was significantly higher (33.33%, 14/42) compared to those with concordant polymicrobial detection in both C&S and PCR (22.22%, 12/54, *p* = 0.041). The RR for CF in patients with missed polymicrobial infections in the C&S-arm was 1.83 [95% CI: 1.18−3.27, *p* = 0.035] compared to those with concordant detection.

Conversely, in the PCR arm, FCl rates in polymicrobial infections (91.67%, 77/84) were comparable to those in monomicrobial infections (85.32%, 93/109, *p* = 0.69). PCR detected (Table 3) a higher proportion of polymicrobial infections compared to C&S (43.52% vs. 31.95%, *p* = 0.033). The FCl rates were also higher in the PCR arm for polymicrobial infections compared to the C&S arm (91.67% vs. 77.78%, *p* = 0.037). The RR for CF in polymicrobial cases was 2.67 [95% CI: 1.12−6.35, *p* = 0.013] higher in the C&S arm than in the PCR arm, indicating a greater likelihood of CF when relying solely on the C&S results.

Among the 23 CF cases in the PCR arm (Table 2), 16 (69.57%) occurred in monomicrobial infections where the PCR and C&S results were concordant for pathogen detection. These cases included *E. coli* (6/16, 37.5%), *Klebsiella pneumoniae* (4/16, 25%), *Pseudomonas aeruginosa* (3/16, 18.75%), and *Enterococcus faecium* (3/16, 18.75%). Additionally, seven (30.43%) CF cases were observed in polymicrobial infections where PCR detected additional pathogens beyond those identified by C&S. Among these cases, co-infections involving *E. coli* and *Pseudomonas aeruginosa* were identified, while *Candida* species were detected alongside *Klebsiella pneumoniae* and *Enterococcus faecium*. Furthermore, infections involving *Pseudomonas aeruginosa* and *Enterococcus faecium* were noted, as well as cases with *E. coli*, *Enterococcus faecium*, and *Candida* spp. Notably, the treatment chosen for each case was appropriate even for the C&S findings, as all pathogens detected by C&S were also identified by PCR.

### 3.3. Missed Pathogens by C&S and Impact on Clinical Outcomes

PCR detected additional pathogens in 54.44% (92/169) of cases in the C&S arm, while C&S missed pathogens in 43.52% (84/193) of cases in the PCR arm. In the C&S arm (Table 4), CF occurred in 28.26% (26/92) of cases where C&S missed pathogens, compared to only 14.29% (11/77) in cases with concordant results (*p* = 0.015). The RR for CF in the missed pathogen cases was 2.16 [95% CI: 1.12−4.16, *p* = 0.010], highlighting the increased risk of CF when relying solely on C&S results. Conversely, in the PCR arm, the CF rate for cases where C&S missed pathogens was lower (not significant) at 8.34% (7/84), compared to 14.68% (16/109) in concordant cases. Patients whose treatment was guided by PCR had better outcomes than those in the C&S arm, where pathogens were missed (91.66% vs. 71.74%, *p* = 0.027), reinforcing the advantage of PCR in optimizing therapy selection.

**Figure 2:**
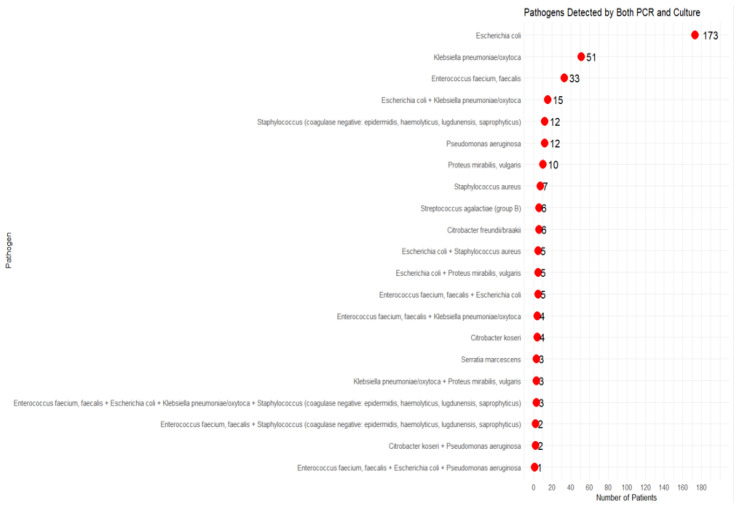
Pathogen detected by both PCR and C&S.

PCR detected all pathogens identified by C&S in all 362 participants (EOS population) and additionally identified numerous pathogens that were not detected by C&S. The distribution of detected pathogens by both the PCR- and C&S arms is presented in Figure 2. *Escherichia coli* was the most frequently identified pathogen, followed by *Klebsiella pneumoniae/oxytoca* and *Enterococcus faecium/faecalis*.

**Figure 3:**
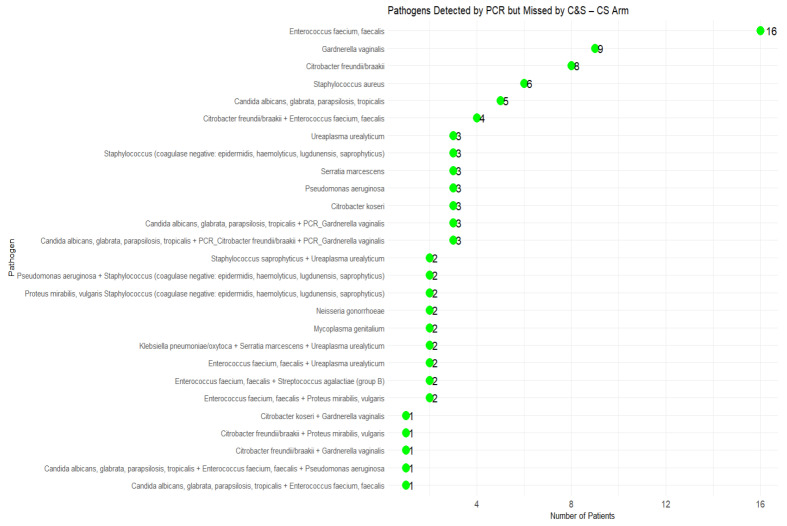
Pathogens Undetected by C&S (C&S Arm).

In the C&S arm, 92 cases (54.44%) had pathogens that were not detected by culture but were identified by PCR (Figure 3). Among these missed pathogens, *Enterococcus faecium/faecalis*, *Gardnerella vaginalis*, and *Candida species* were the most frequently overlooked organisms. Additionally, several polymicrobial infections involving *Citrobacter* spp., *Mycoplasma genitalium*, and *Neisseria gonorrhoeae* were not detected by C&S.

**Figure 4:**
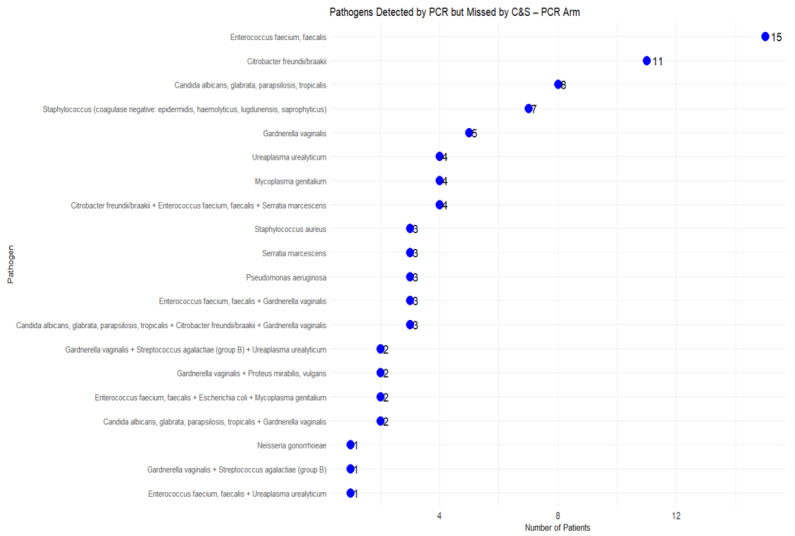
Pathogens Undetected by C&S (PCR arm).

Similarly, in the PCR arm, 84 cases (43.52%) had pathogens detected by PCR that were not identified by C&S (Figure 4). Frequently missed pathogens included *Candida species*, *Enterococcus faecium/faecalis*, and *Gardnerella vaginalis*. Furthermore, *Mycoplasma genitalium*, *Ureaplasma urealyticum*, and *Neisseria gonorrhoeae*, were overlooked, suggesting that C&S methods may be inadequate for detecting certain fastidious or slow-growing pathogens due to their complex growth requirements or slow growth rates in culture media.

Table 5 presents patient-level cases where PCR (in the PCR arm) detected additional pathogens that were missed by standard C&S, demonstrating its impact on antimicrobial selection and clinical outcomes. In each case, PCR-guided therapy ensured appropriate treatment of all detected pathogens, whereas culture-based treatment alone would have been inadequate, potentially resulting in therapeutic failure or suboptimal patient outcomes.

Table 6 summarizes patient-level cases where PCR (in the C&S arm) detected additional pathogens that were missed by culture-based diagnostics, contributing to CF. In each case, standard C&S identified a primary uropathogen, and antibiotic therapy was selected accordingly. However, PCR revealed additional co-infecting organisms, including *Candida species*, *Pseudomonas aeruginosa*, *Enterococcus faecium/faecalis*, and *Serratia marcescens*, which were not covered by the initial therapy. PCR data availability would have enabled alternative or adjunctive therapy (e.g., fluoroquinolones for *Pseudomonas*, fluconazole for *Candida spp*., and cephalexin for *Proteus mirabilis*), leading to improved clinical outcomes.

### 3.4. Undetected Phenotypic Resistance by C&S and Its Impact on Antibiotic Selection and Clinical Outcomes

Table 7 and Table 8 summarize the resistance profiles detected by PCR-based molecular testing and C&S testing in 362 cases of cUTI. Table 07 presents 780 genetic resistance markers identified via PCR, including those conferring resistance to beta-lactams (e.g., blaKPC, blaCTX-M, and blaOXA−48), fluoroquinolones (qnr genes), aminoglycosides, and glycopeptides (VanA/VanB genes). Resistance markers with strong phenotypic correlation (e.g., blaKPC, mecA) were deemed definitive indicators of resistance, while those with less consistent correlation (e.g., qnr genes) were subject to clinical evaluation. Table 8 reports 614 cases of phenotypic resistance detected through C&S, with ampicillin (168 cases), trimethoprim-sulfamethoxazole (146 cases), and tigecycline (127 cases) resistance being the most frequent. Resistance to carbapenems (28 cases), cephalosporins (41 cases), and fluoroquinolones (39 cases) was also observed.

To assess the clinical impact of undetected phenotypic resistance by C&S, we compared CF rates between cases where C&S failed to detect phenotypic resistance and cases where resistance was accurately detected by either C&S or PCR. In the C&S arm, 42 cases had undetected phenotypic resistance, whereas 121 cases had concordant resistance detection. In the PCR arm, 48 cases had undetected phenotypic resistance, while 165 cases had concordant resistance detection. Importantly, the genetic resistance markers detected by PCR were associated both with concordantly detected pathogens and with pathogens missed by C&S but identified via molecular testing.

Table 9 summarizes CF rates in these groups. Failure was significantly higher in the C&S arm when phenotypic resistance was undetected (50%) compared to cases where resistance was concordantly identified (13.22%, *p* = 0.001). In contrast, the PCR arm had a lower failure rate of 12.5% in undetected phenotypic resistance cases and 10.98% in concordant detection cases. The RR of CF due to undetected resistance in the C&S arm was 2.31 [95% CI: 1.07–3.75, *p* = 0.014] compared to concordant detection cases, reinforcing that C&S-based therapy is at a higher risk for CF when phenotypic resistance is not identified.

Table 10 presents 16 cUTIs cases where C&S testing failed to detect key resistance mechanisms, resulting in inappropriate antibiotic selection and increased CF risk. These cases are categorized into two groups:

The first group consists of cases where phenotypic resistance was missed despite successful pathogen identification (*n* = 8). Culture identified the primary pathogen, but sensitivity testing failed to recognize resistance mechanisms such as fluoroquinolone resistance (qnr genes), extended-spectrum β-lactamases (CTX-M, SHV), carbapenemases (OXA, KPC, and VIM), and vancomycin resistance (VanA and VanB). As a result, antibiotics like fluoroquinolones, cephalosporins, and aminoglycosides were often prescribed inappropriately, leading to a need for alternative therapies such as carbapenems, fosfomycin, or linezolid.

The second group includes cases where culture missed a co-pathogen with clinically significant resistance (*n* = 8). PCR identified additional resistant pathogens, including *Candida spp.* (azole resistance), *Enterococcus spp.* (vancomycin resistance), and *Pseudomonas aeruginosa* (carbapenemases), which were not recovered by C&S. The lack of detection of these resistant co-pathogens resulted in inadequate antibiotic coverage, particularly when β-lactams or fluoroquinolones were prescribed without appropriate antifungal or anti-VRE therapies.

Table 11 presents 17 cases where PCR detected resistance markers or additional pathogens that were missed by culture-based testing, guiding more effective antibiotic selection and preventing potential CF. Among these cases, 10 involved polymicrobial infections, where C&S failed to isolate all clinically relevant pathogens identified by PCR. Notably, *Enterococcus faecalis, Pseudomonas aeruginosa, Proteus mirabilis, Staphylococcus aureus,* and *Candida spp.* were frequently missed in the presence of dominant Gram-negative bacteria such as *Escherichia coli* and *Klebsiella pneumoniae*. In these cases, PCR-guided therapy ensured adequate antimicrobial coverage, avoiding the inadequate pathogen targeting of culture-based treatment alone.

In all 17 cases, PCR identified resistance genes that culture-based phenotypic testing failed to detect or fully characterize. These included carbapenemases (*OXA−48, IMP*), extended-spectrum beta-lactamases (ESBLs) cephalosporinases (*CTX-M, AmpC*), fluoroquinolone resistance determinants (*qnrB, qnrS*), and vancomycin resistance genes (*VanA, VanB*). Importantly, case 17 highlighted the challenge of vancomycin-resistant Enterococcus faecalis (VanB) in a polymicrobial infection, which was missed by C&S but correctly identified by PCR, enabling the selection of fosfomycin instead of an ineffective beta-lactam regimen.

## 4. Discussion

### 4.1. Test Performance Across Clinical and Demographic Subpopulations

The findings of this study reaffirm the high sensitivity of PCR-based diagnostics in detecting culture-confirmed infections across diverse clinical and demographic subpopulations (Table 1). PCR identified most infections confirmed by conventional C&S testing in both the enrolled population (95.32%) and the EOS population (88.06%), underscoring its robust capability in pathogen detection and its potential to reduce missed clinically significant infections.

Specificity was lower, particularly in the enrolled population (~38%), due to the detection of additional pathogens by PCR not recovered by C&S, leading to more PCR-positive but C&S-negative cases. These discordances were further explored in Part II of this study [5]. At EOS, specificity improved to ~63% across all subgroups, suggesting that initial discrepancies may diminish over time due to treatment effects, changes in infection dynamics, or improved understanding of viability in culture.

Sensitivity and specificity varied slightly across study sites, age groups, sex, symptom burden, and infection complexity. Notably, the upward trend in specificity at EOS reinforces that PCR-positive, C&S-negative results may still reflect true infections. Culture methods often fail to detect viable but non-culturable organisms, leading to underestimation of infection prevalence [6].

An emerging body of literature has explored the role of urinary biomarkers, such as neutrophil gelatinase-associated lipocalin (NGAL), interleukin−8 (IL−8), and interleukin−1 beta (IL−1β), in elucidating the limitations of culture-based diagnostics. Multiple studies have demonstrated that patients with PCR-positive but C&S-negative results exhibit elevated levels of these biomarkers, supporting the hypothesis that PCR is detecting true infections overlooked by culture-based methods [6,7,8,9]. These findings suggest that the lower specificity of PCR relative to C&S does not necessarily indicate overdiagnosis but may instead highlight the shortcomings of conventional culture in capturing the full spectrum of uropathogens involved in cUTIs.

The clinical significance of PCR-detected pathogens remains a crucial consideration. All study participants had symptomatic cUTIs with underlying risk factors, reinforcing that test results must be interpreted alongside the clinical presentation. PCR’s consistent detection accuracy, particularly where C&S failed, warrants further evaluation concerning patient outcomes.

Overall, these findings support PCR as a highly sensitive diagnostic tool and emphasize the importance of integrating molecular diagnostics with clinical assessment rather than relying solely on conventional culture to confirm infection.

### 4.2. Polymicrobial Detection and Impact on Clinical Outcomes

The results of this study indicate that polymicrobial infections are a critical factor influencing FCls in cUTIs (Table 2). In the C&S arm, the failure to detect polymicrobial infections was associated with a significantly higher CF rate (33.33%, 14/42) compared to cases where both C&S and PCR concorded polymicrobial infections (22.22%, 12/54, *p* = 0.041). This finding suggests that the limitations of C&S in detecting polymicrobial infections contributed to CF, potentially due to incomplete pathogen coverage in empiric and definitive therapy.

In contrast, optimized therapy and prevention of CF were achieved in the PCR arm, as evidenced by comparable FCl rates between polymicrobial infections (91.67%, 77/84) and monomicrobial infections (85.32%, 93/109, *p* = 0.69), demonstrating the impact of comprehensive pathogen detection (Table 3). These findings underscore the potential benefit of PCR-based diagnostics in improving clinical outcomes by providing a more comprehensive microbial profile at the time of treatment decision-making.

Notably, PCR detected a significantly higher proportion of polymicrobial infections compared to C&S (43.52% vs. 31.95%, *p* = 0.033). The RR for CF in polymicrobial cases was 2.67 [95% CI: 1.12−6.35, *p* = 0.013] higher in the C&S arm than in the PCR arm, indicating that reliance on culture-based diagnostics may contribute to suboptimal treatment outcomes in polymicrobial infections.

These findings align with previous studies that have consistently demonstrated the superiority of molecular diagnostic tests, such as PCR, in detecting polymicrobial infections compared to conventional urine culture methods. However, concerns regarding their lower specificity remain [10,11,12]. While C&S remains the standard for antimicrobial sensitivity testing, its limitations in pathogen recovery, particularly in polymicrobial infections, suggest that incorporating molecular diagnostics into routine clinical practice may enhance treatment efficacy and reduce failure rates.

Further studies are needed to evaluate the long-term clinical implications of PCR-guided therapy, particularly about antimicrobial stewardship and resistance development. Nonetheless, the current findings strongly support the integration of PCR testing in the diagnostic workflow for cUTIs, particularly in patients at high risk for polymicrobial infections.

Several factors may have contributed to the 23 clinical failure (CF) cases in the PCR arm (Table 2), including polymicrobial complexity, intrinsic resistance, biofilm formation, fungal persistence, and host-related factors such as immunosuppression and comorbidities. Polymicrobial infections pose treatment challenges as interspecies interactions, particularly between bacterial and fungal pathogens, can enhance virulence and antimicrobial resistance. Synergistic effects—such as those between *E. coli* and *Pseudomonas aeruginosa*—may increase immune evasion and inflammation, reducing treatment efficacy [1,2,3].

Intrinsically resistant organisms like *Pseudomonas aeruginosa* and *Enterococcus faecium* likely contributed to poor outcomes, particularly in recurrent cases with inadequate initial antimicrobial coverage [1,7]. Biofilm formation and fungal persistence further hinder treatment, as pathogens such as *Pseudomonas aeruginosa*, *Enterococcus faecium*, and *Candida spp.* are known biofilm producers that resist immune responses and antibiotic penetration [3]. Fungal co-infections—especially involving *Candida spp.*—may worsen outcomes by modulating bacterial behavior and enhancing biofilm development, as seen in co-infections with *Klebsiella pneumoniae* and *Enterococcus faecium* [12,13].

Host factors such as immunosuppression may also impair infection clearance despite appropriate therapy. These findings underscore the need for targeted strategies addressing polymicrobial dynamics, biofilm-related resistance, fungal persistence, and host vulnerability. PCR’s ability to rapidly detect such complex infections supports its role in guiding more effective, individualized treatment approaches in cUTI management [1,3].

### 4.3. Pathogens Missed by C&S and Impact on Clinical Outcomes

PCR detected additional pathogens in 54.44% (92/169) of cases in the C&S arm, while C&S missed pathogens in 43.52% (84/193) of cases in the PCR arm. The failure of C&S to detect key pathogens more frequently than PCR in both arms underscores the limitations of traditional culture-based diagnostics.

Patients in the C&S arm with missed pathogens had significantly higher CF rates (Table 2). Specifically, 70.27% (26/37) of all CF in the C&S-arm were linked to cases where C&S missed pathogens, compared to only 30.43% (7/23) of CF in the PCR-arm. This indicates that incomplete pathogen detection by C&S was a major contributor to CF, whereas PCR-based detection improved targeted therapy and clinical success.

The findings from Table 5 and Table 6 emphasize the clinical relevance of polymicrobial infections in cUTI, particularly the presence of fungal co-infections that were overlooked by C&S. PCR-based diagnostics played a crucial role in preventing CF by enabling faster, more precise, and comprehensive antimicrobial regimen selection. The results further underscore the limitations of culture-based methods and support the integration of molecular diagnostics for more effective infection management.

Traditional urine C&S methods tend to favor *Escherichia coli* growth, often missing fastidious organisms and frequently failing to identify more than two pathogens in polymicrobial infections, leading to incomplete or inaccurate diagnoses. This limitation is compounded by previous data suggesting that urine C&S detects only around 60% of acute UTI cases, leaving many infections undiagnosed [7,12,13]. PCR methods, with their superior sensitivity and reduced false-negative rate, surpass conventional C&S methods by detecting a wider range of pathogens, including those often missed by culture, thus significantly reducing the likelihood of missed diagnoses.

Furthermore, traditional C&S methods often favor the growth of a single fast-growing pathogen, which leads to the selection of a predominant clone and may obscure the presence of co-infections or multi-resistant subpopulations [14]. In contrast, PCR-based diagnostics can reliably detect co-pathogens—such as Enterococcus spp. and Citrobacter spp.—and capture multi-resistant subpopulations by analyzing DNA directly from urine samples. This methodological difference may account for cases where therapy guided by PCR results yields improved outcomes compared to decisions based solely on traditional AST. These findings support the integrated use of molecular diagnostics alongside standard culture methods to enhance overall pathogen detection and optimize antimicrobial selection in cUTI management.

### 4.4. Undetected Phenotypic Resistance by C&S and Its Impact on Antibiotic Selection and Clinical Outcomes

Table 7 and Table 8 summarize the resistance profiles detected by PCR-based molecular testing (780 genetic resistance markers identified via PCR) and C&S (614 cases of phenotypic resistance detected through C&S) in 362 cases of cUTI. The discrepancy between genetic and phenotypic resistance detection influenced treatment decisions, particularly in the PCR group, where all detected organisms were treated equally. The higher rate of polymicrobial detection in PCR cases led to the use of broader-spectrum antibiotics, whereas culture-based treatment was limited to phenotypically resistant pathogens. This marked difference can be partly attributed to the inherent limitations of culture-based methods, which assess a limited number of colonies and are unable to capture heteroresistant populations. PCR, by analyzing the entire microbial community within a sample, is capable of detecting subpopulations harboring resistance determinants that may be missed during clonal selection in C&S. Such heteroresistance can significantly contribute to therapeutic failure, as highlighted by Band and Weiss [15] and further elaborated by Xu et al. [16]. These observations underscore that PCR not only provides a comprehensive resistance profile but also has the potential to guide more effective antimicrobial therapy by revealing hidden heteroresistant subpopulations—a critical advantage over conventional C&S approaches.

Of the 37 total CFs in the C&S arm, 23 (62.16%) were due to undetected phenotypic resistance, emphasizing the clinical consequences of resistance misclassification. In contrast, in the PCR arm, only 6 of 23 failures (26.07%) were linked to undetected phenotypic resistance by C&S, suggesting a lower risk of CF when genetic resistance markers guide therapy.

Across all cases (Table 10), the failure of C&S to detect resistance mechanisms was associated with CF and the need for broader-spectrum or alternative antibiotics such as ceftazidime-avibactam, colistin, and echinocandins. These findings highlight the limitations of traditional phenotypic resistance testing in cUTIs and support the integration of molecular diagnostics to improve antimicrobial therapy selection.

In Table 11, while phenotypic resistance testing generally aligned with PCR findings, it was limited in detecting resistance mediated by efflux pumps and low-level beta-lactamase expression, particularly in *Pseudomonas aeruginosa* and *Enterobacterales*. PCR’s ability to provide a comprehensive resistance profile and uncover cryptic polymicrobial infections underscores its clinical value in guiding optimized, targeted therapy and improving treatment outcomes in cUTIs.

Several studies [14,17,18,19] have shown that metagenomic sequencing and multiplex PCR outperform conventional culture and sensitivity (C&S) by detecting resistance genes and co-pathogens often missed in standard testing. PCR enables rapid identification of critical resistance determinants—including ESBLs, carbapenemases, and vancomycin resistance—that may not be phenotypically expressed in vitro but contribute to treatment failure under selective pressure [20,21,22,23]. Additionally, C&S often under-detects organisms like *Enterococcus faecalis*, *Proteus mirabilis*, and *Pseudomonas aeruginosa* during polymicrobial infections, whereas PCR more reliably identifies these pathogens and associated resistance markers such as VanB or qnr genes. This facilitates more accurate therapy selection, as in cases where PCR detection of *Klebsiella pneumoniae* and *E. faecalis* led to fosfomycin use instead of inappropriate beta-lactams. Moreover, phenotypic sensitivity testing is limited by selective gene expression, which can result in in vitro sensitivity despite clinical resistance [23]. PCR, being genotypic, overcomes this limitation and provides a more comprehensive resistance profile. These findings support integrating molecular diagnostics with traditional phenotypic methods to enhance resistance detection, guide more effective therapy, and reduce clinical failures in cUTI management.

## 5. Conclusions

Overall, our findings confirm the crucial role of molecular diagnostics, particularly PCR—in enhancing the detection and management of complicated urinary tract infections (cUTIs). In comparison to conventional C&S testing, PCR demonstrated superior sensitivity for pathogen identification, effectively detecting polymicrobial infections and fastidious or slow-growing organisms that are frequently missed by traditional culture methods. This enhanced detection capability was associated with improved favorable clinical outcomes (FCl), especially in cases of polymicrobial infection where incomplete detection by C&S correlated with higher rates of clinical failure (CF).

In addition, PCR showed significant advantages in identifying antimicrobial resistance markers, thereby equipping clinicians with the necessary data for early and targeted antibiotic therapy. Our results indicate that undetected phenotypic resistance and hetero-resistance due to limited clonal analysis via conventional C&S methods are major contributors to CF, whereas PCR-guided management is associated with a lower therapeutic failure rate.

These findings strongly support integrating PCR-based diagnostics with standard C&S methods to achieve a comprehensive assessment of microbial and resistance profiles in cUTIs. By providing rapid and precise pathogen and resistance gene identification, PCR facilitates earlier and more effective antimicrobial treatment, which can ultimately reduce clinical failure and enhance antimicrobial stewardship efforts.

## Figures and Tables

**Table 1:** Analysis of Test Performance in Relevant Clinical and Demographic Subpopulations.

		Enrolled Population (*n* = 773)		EOS Population (*n* = 362)	
		Sensitivity	Specificity	Sensitivity	Specificity
Sites	AUG	94.37%	40.23%	94.16%	63.25%
	ALB	96.12%	36.24%	85.62%	62.88%
	NRM	92.66%	38.15%	87.51%	62.95%
	PSJ	97.25%	39.45%	88.22%	64.55%
	SCV	95.24%	38.12%	91.35%	61.22%
	SET	94.66%	37.69%	94.23%	61.35%
Age	Elderly (≥65 yrs)	94.47%	38.35%	85.12%	62.45%
	Non-Elderly (<65 yrs)	96.32%	39.12%	88.74%	63.88%
Sex at birth	Male	95.71%	37.23%	86.72%	61.58%
	Female	95.42%	39.47%	84.71%	64.25%
cUTI Symptoms’ association	02 Symptoms	94.50%	37.55%	87.18%	62.22%
	03 Symptoms	95.43%	38.73%	85.23%	62.57%
	04 Symptoms	95.83%	38.66%	98.33%	63.45%
cUTI event	Mono-Infection	95.12%	38.56%	88.23%	62.55%
	Poly-Infection	96.66%	37.89%	87.57%	63.89%
Overall		95.32%	38.30%	88.06%	62.91%

**Table 2:** Impact of Polymicrobial vs. Monomicrobial Infections on Clinical Outcomes.

C&S Arm (*n* = 169)			PCR Arm (*n* = 193)		
Pathogen detection	CF	FCl	Pathogen detection	CF	FCl
Mono C&S (1 Pathogen) (*n* = 115)	25	90	Mono PCR (1 Pathogen) (*n* = 109)	16	93
PCR 1 pathogen (*n* = 73)	11	62	C&S 1 pathogens (*n* = 109)	16	93
PCR 2 pathogens (*n* = 42)	14	28	Poly PCR (2 Pathogens) (*n* = 34)	0	34
Poly C&S (2 Pathogens) (*n* = 34)	8	26	C&S 1 pathogens (*n* = 34)	0	34
PCR 3 pathogens (*n* = 17)	3	14	Poly PCR (3 Pathogens) (*n* = 22)	3	19
PCR 4 pathogens (*n* = 15)	4	11	C&S 1 pathogens (*n* = 9)	1	8
PCR 5 pathogens (*n* = 2)	1	1	C&S 2 pathogens (*n* = 13)	2	11
Poly C&S (3 Pathogens) (*n* = 20)	4	16	Poly PCR (4 Pathogens) (*n* = 21)	3	18
PCR 3 pathogens (*n* = 4)	0	4	C&S 2 pathogens (*n* = 11)	3	8
PCR 4 pathogens (*n* = 16)	4	12	C&S 3 pathogens (*n* = 10)	0	10
Total (*n* = 169)	37	132	Poly PCR (5 Pathogens) (*n* = 7)	1	6
			C&S 3 pathogens (*n* = 7)	1	6
			Total (*n* = 193)	23	170

**Table 3:** Association Between Infection Type and FCl in Each Arm.

	Monomicrobial Cases		Polymicrobial Cases	
	Detection	FCl	Detection	FCl
C&S Arm	115 (68.05%)	90 (78.26%)	54 (31.95%) *	42 (77.78%) ^#^
PCR Arm	109 (56.48%)	93 (85.32%)	84 (43.52%) *	77 (91.67%) ^#^

* Significance difference (*p* < 0.05) / ^#^ Significance difference (*p* < 0.05)

**Table 4:** Comparison of CF Rates Based on C&S Pathogen Detection Accuracy.

	CF in Missed Cases	CF in Concordant Cases
**C&S Arm**	26/92 (28.26%) *	11/77 (14.29%) *
**PCR Arm**	7/84 (8.34%)	16/109 (14.68%)

* Significance difference (*p* < 0.05).

**Table 5:** PCR-Detected, Culture-Missed Pathogens: Impact on Treatment Decision and Clinical Outcomes.

Case	Pathogen(s) Detected by PCR	Treatment Based on PCR Results	Outcome of PCR-Guided Treatment	Pathogen(s) Detected by Culture	Pathogen(s) Detected by PCR but Missed by Culture	Treatment Chosen if It Was Based on Culture Results Only	Risk of Treatment Failure if Guided by CS?
1	*Escherichia coli + Enterococcus faecalis*	Nitrofurantoin	FCl	*Escherichia coli*	*Enterococcus faecalis*	Cephalexin	High
2	*Klebsiella pneumoniae + Pseudomonas aeruginosa*	Cefepime	FCl	*Klebsiella pneumoniae*	*Pseudomonas aeruginosa*	Amoxicillin/Clavulanate	High
3	*Escherichia coli + Proteus mirabilis*	Ciprofloxacin	FCl	*Escherichia coli*	*Proteus mirabilis*	Nitrofurantoin	High
4	*Enterococcus faecalis + Klebsiella pneumoniae*	Nitrofurantoin + Amoxicillin/Clavulanate	FCl	*Klebsiella pneumoniae*	*Enterococcus faecalis*	Nitrofurantoin	High
5	*Escherichia coli + Serratia marcescens*	Ciprofloxacin	FCl	*Escherichia coli*	*Serratia marcescens*	Nitrofurantoin	High
6	*Enterococcus faecalis + Staphylococcus aureus*	Amoxicillin/Clavulanate	FCl	*Enterococcus faecalis*	*Staphylococcus aureus*	Nitrofurantoin	High
7	*Proteus mirabilis + Klebsiella pneumoniae*	Ceftriaxone	FCl	*Klebsiella pneumoniae*	*Proteus mirabilis*	Amoxicillin/Clavulanate	High
8	*Escherichia coli + Staphylococcus aureus*	Trimethoprim/Sulfamethoxazole	FCl	*Escherichia coli*	*Staphylococcus aureus*	Nitrofurantoin	High
9	*Klebsiella pneumoniae + Escherichia coli + Enterococcus faecalis*	Fosfomycin	FCl	*Klebsiella pneumoniae + Escherichia coli*	*Enterococcus faecalis*	Amoxicillin/Clavulanate	High
10	*Pseudomonas aeruginosa + Proteus mirabilis*	Ciprofloxacin	FCl	*Proteus mirabilis*	*Pseudomonas aeruginosa*	Trimethoprim/Sulfamethoxazole	High
11	*Serratia marcescens + Escherichia coli*	Ciprofloxacin	FCl	*Escherichia coli*	*Serratia marcescens*	Nitrofurantoin	High
12	*Enterococcus faecalis + Klebsiella pneumoniae*	Amoxicillin/Clavulanate	FCl	*Enterococcus faecalis*	*Klebsiella pneumoniae*	Nitrofurantoin	High
13	*Escherichia coli + Staphylococcus aureus*	Cephalexin	FCl	*Escherichia coli*	*Staphylococcus aureus*	Nitrofurantoin	High
14	*Enterococcus faecalis + Escherichia coli*	Nitrofurantoin	FCl	*Escherichia coli*	*Enterococcus faecalis*	Cephalexin	High
15	*Proteus mirabilis + Serratia marcescens*	Ciprofloxacin	FCl	*Proteus mirabilis*	*Serratia marcescens*	Trimethoprim/Sulfamethoxazole	High
16	*Klebsiella pneumoniae + Pseudomonas aeruginosa + Escherichia coli*	Cefepime	FCl	*Klebsiella pneumoniae + Escherichia coli*	*Pseudomonas aeruginosa*	Amoxicillin/Clavulanate	High
17	*Enterococcus faecalis + Staphylococcus aureus + Escherichia coli*	Nitrofurantoin + Cephalexin	FCl	*Escherichia coli*	*Enterococcus faecalis + Staphylococcus aureus*	Trimethoprim/Sulfamethoxazole	High
18	*Proteus mirabilis + Escherichia coli*	Trimethoprim/Sulfamethoxazole	FCl	*Escherichia coli*	*Proteus mirabilis*	Nitrofurantoin	High
19	*Klebsiella pneumoniae + Candida* spp.	Meropenem + Fluconazole	FCl	*Klebsiella pneumoniae*	*Candida*	Meropenem (no antifungal)	High
20	*Enterococcus faecium + Candida* spp.	Linezolid + Fluconazole	FCl	*Enterococcus faecium*	*Candida*	Linezolid (no antifungal)	High
21	*Escherichia coli + Candida* spp.	Ciprofloxacin + Fluconazole	FCl	*Escherichia coli*	*Candida*	Ciprofloxacin (no antifungal)	High
22	*Pseudomonas aeruginosa + Candida* spp.	Meropenem + Fluconazole	FCl	*Pseudomonas aeruginosa*	*Candida*	Meropenem (no antifungal)	High

**Table 6:** Impact of C&S-Based Treatment: Clinical Failure and Alternative Therapy for PCR-Detected, C&S-Missed Pathogens.

Case	Pathogen(s) Found in Culture (Guided Treatment)	Antibiotic Prescribed Based on Culture	Outcome of C&S Guided Treatment	Pathogen Detected by PCR	Pathogen(s) Detected by PCR but Missed by Culture	Alternative Antibiotic if Treatment Was Guided by PCR
1	*Escherichia coli*	Ciprofloxacin	CF	*Enterococcus faecium/faecalis + Escherichia coli*	*Enterococcus faecium/faecalis*	Fosfomycin or Nitrofurantoin
2	*Escherichia coli*	Nitrofurantoin	CF	*Pseudomonas aeruginosa + Escherichia coli*	*Pseudomonas aeruginosa*	Ciprofloxacin or Levofloxacin
3	*Enterococcus faecium/faecalis*	Nitrofurantoin	CF	*Escherichia coli + Klebsiella pneumoniae + Enterococcus faecium/faecalis*	*Escherichia coli + Klebsiella pneumoniae*	Ciprofloxacin or Trimethoprim/Sulfamethoxazole
4	*Escherichia coli*	Nitrofurantoin	CF	*Proteus mirabilis + Escherichia coli*	*Proteus mirabilis*	Ciprofloxacin or Cephalexin
5	*Escherichia coli*	Fosfomycin	CF	*Serratia marcescens + Escherichia coli*	*Serratia marcescens*	Ciprofloxacin
6	*Escherichia coli*	Cefepime	CF	*Escherichia coli + Candida* spp.	*Candida* spp.	+ Fluconazole
7	*Klebsiella pneumoniae*	Ciprofloxacin	CF	*Enterococcus faecium/faecalis + Klebsiella pneumoniae*	*Enterococcus faecium/faecalis*	Nitrofurantoin or Fosfomycin
8	*Klebsiella pneumoniae*	Nitrofurantoin	CF	*Pseudomonas aeruginosa + Klebsiella pneumoniae*	*Pseudomonas aeruginosa*	Ciprofloxacin or Levofloxacin
9	*Escherichia coli*	Trimethoprim/Sulfamethoxazole	CF	*Staphylococcus aureus + Escherichia coli*	*Staphylococcus aureus*	Cephalexin or Doxycycline
10	*Proteus mirabilis*	Ciprofloxacin	CF	*Enterococcus faecium/faecalis + Proteus mirabilis*	*Enterococcus faecium/faecalis*	Nitrofurantoin or Fosfomycin
11	*Klebsiella pneumoniae*	Nitrofurantoin	CF	*Proteus mirabilis + Klebsiella pneumoniae*	*Proteus mirabilis*	Ciprofloxacin or Cephalexin
12	*Escherichia coli*	Trimethoprim/Sulfamethoxazole	CF	*Candida glabrata + Escherichia coli*	*Candida* spp.	+ Fluconazole
13	*Escherichia coli*	Ciprofloxacin	CF	*Candida* spp. *+ Enterococcus faecium + Escherichia coli*	*Candida* spp. *+ Enterococcus faecium*	+ Fluconazole
14	*Klebsiella pneumoniae*	Nitrofurantoin	CF	*Escherichia coli + Pseudomonas aeruginosa + Klebsiella pneumoniae*	*Escherichia coli + Pseudomonas aeruginosa*	Ciprofloxacin or Levofloxacin
15	*Proteus mirabilis*	Ciprofloxacin	CF	*Proteus mirabilis + Serratia marcescens*	*Serratia marcescens*	Cefepime
16	*Escherichia coli*	Cefepime	CF	*Escherichia coli + Candida* spp.	*Candida* spp.	+ Fluconazole
17	*Klebsiella pneumoniae*	Nitrofurantoin	CF	*Candida krusei + Enterococcus faecium + Klebsiella pneumoniae*	*Candida spp + Enterococcus faecium*	Caspofungin
18	*Escherichia coli*	Nitrofurantoin	CF	*Pseudomonas aeruginosa + Serratia marcescens + Escherichia coli*	*Pseudomonas aeruginosa + Serratia marcescens*	Ciprofloxacin or Levofloxacin
19	*Escherichia coli*	Nitrofurantoin	CF	*Enterococcus faecium + Candida albicans + Escherichia coli*	*Enterococcus faecium + Candida* spp.	+ Fluconazole
20	*Klebsiella pneumoniae + Escherichia coli*	Ciprofloxacin	CF	*Enterococcus faecium/faecalis + Klebsiella pneumoniae + Escherichia coli*	*Enterococcus faecium/faecalis*	Fosfomycin or Nitrofurantoin
21	*Klebsiella pneumoniae*	Ciprofloxacin	CF	*Candida parapsilosis + Pseudomonas aeruginosa + Klebsiella pneumoniae*	*Candida* spp. *+ Pseudomonas aeruginosa*	+ Fluconazole
22	*Klebsiella pneumoniae*	Nitrofurantoin	CF	*Escherichia coli + Proteus mirabilis + Klebsiella pneumoniae*	*Escherichia coli + Proteus mirabilis*	Ciprofloxacin or Cephalexin
23	*Staphylococcus aureus*	Vancomycin	CF	*Staphylococcus aureus + Enterococcus faecium*	*Enterococcus faecium*	Linezolid or Daptomycin
24	*Escherichia coli*	Ciprofloxacin	CF	*Candida tropicalis + Enterococcus faecium + Escherichia coli*	*Candida* spp. *+ Enterococcus faecium*	+ Fluconazole
25	*Proteus mirabilis*	Ciprofloxacin	CF	*Proteus mirabilis + Klebsiella pneumoniae*	*Klebsiella pneumoniae*	Cephalexin
26	*Enterococcus faecium/faecalis*	Nitrofurantoin	CF	*Escherichia coli + Klebsiella pneumoniae + Enterococcus faecium/faecalis*	*Escherichia coli + Klebsiella pneumoniae*	Ciprofloxacin or Trimethoprim/Sulfamethoxazole

**Table 7:** Number of cases of detected genetic resistance markers (*n* = 362).

Resistance	Number of Cases	Affected Antibiotic Class
ACT, MIR, FOX, ACC Groups (Beta Lactams)	32	Cephalosporins (e.g., cefotaxime, ceftazidime), penicillins, monobactams
Class A ß-lactamase; blaKPC	3	Carbapenems (e.g., meropenem, imipenem, ertapenem), cephalosporins, penicillins
Class A ß-lactamase; CTX-M-Group1	29	Cephalosporins (e.g., ceftriaxone, ceftazidime, cefotaxime), penicillins
Class B metallo-ß-lactamase; blaNDM	3	Carbapenems, cephalosporins, penicillins
Class D oxacillinase OXA−48	11	Carbapenems (e.g., imipenem, meropenem), penicillins
Class D oxacillinase OXA-−51	3	
dfr (A1, A5), sul (1,2) probes (Sulfamethoxazole and trimethoprim)	142	Diamino-pyrimidine (Trimethoprim), Sulfonamide, trimethoprim-sulfamethoxazole
ermB, C; mefA	172	Macrolides (e.g., erythromycin, azithromycin, clarithromycin), lincosamides (clindamycin)
IMP, NDM, VIM Groups (Carbapenem)	20	Carbapenems, cephalosporins, penicillins
MRSA* Mec-A gene	86	Beta-lactams, including methicillin, oxacillin, penicillins, and cephalosporins
PER−1/VEB−1/GES−1 Groups (ESBL)	6	Cephalosporins, penicillins, monobactams
qnrA1, A2	1	Fluoroquinolones (e.g., ciprofloxacin, levofloxacin)
qnrB	36	
qnrS	10	
tetB, tetM	220	Tetracyclines (e.g., doxycycline, minocycline, tetracycline)
VanA, VanB (Vancomycin)	6	Glycopeptide (Vancomycin, Teicoplanin)
Total Genetic Resistance Detected	**780**	

**Table 8:** Number of cases of detected phenotypic resistance (*n* = 362).

Antibiotic Groups	Phenotypical Resistance Cases
Ampicillin/Ampicillin–Sulbactam	168
Aztreonam/Cefazolin/Cefepime/Cefoxitin/Ceftazidime/Ceftriaxone	41
Ciprofloxacin/Levofloxacin	39
Ertapenem/Imipenem/Meropenem	28
Gentamicin/Tobramycin/Amikacin	52
Nitrofurantoin	13
Tigecycline	127
Trimethoprim/Sulfamethoxazole (TMP-SMX)	146
Total Phenotypical Resistance Detected	614

**Table 9:** CF Rates in Cases with Undetected Phenotypic Resistance.

	CF for Cases with Undetected Phenotypic Resistance (with the Presence of Their Molecular Equivalent)	CF with Concordant Resistance Detection Cases (C&S and PCR)
C&S Arm	16/42 (50%) *	21/121 (13.22%) *
PCR Arm	6/48 (12.5%)	17/155 (10.98%)

* Significance difference (*p* < 0.05).

**Table 10:** Clinical Impact: C&S Missed Resistance, PCR-Detected Genes, and Inappropriate Antibiotic Therapy (C&S Arm).

Case	Pathogen(s) Detected by PCR (with Resistance Marker)	Pathogen(s) Found in Culture	Phenotypic Resistance Detected in Culture	Antibiotic Prescribed Based on Culture	Missed Pathogen(s) by Culture	Undetected Resistance in Missed Pathogen	Outcome of C&S Guided Treatment	Alternative/Additional Treatment Required?
1	*Escherichia coli* (qnrB, blaCTX-M−15)	*Escherichia coli*	None detected	Ciprofloxacin	*Enterococcus faecium*	VanB (VRE)	CF	Linezolid or Daptomycin
2	*Klebsiella pneumoniae* (KPC−2)	*Klebsiella pneumoniae*	Fluoroquinolones, Aminoglycosides	Cefepime	*Pseudomonas aeruginosa*	OXA−51 (Carbapenemase)	CF	Ceftazidime-Avibactam or Cefiderocol
3	*Enterococcus faecium* (VanA)	*Enterococcus faecium*	Tetracyclines	Ampicillin	*Klebsiella pneumoniae*	CTX-M−15 (ESBL)	CF	Ceftazidime-Avibactam or Meropenem
4	*Staphylococcus aureus* (mecA)	*Staphylococcus aureus*	Macrolides, Aminoglycosides	Clindamycin	*Escherichia coli*	qnrB1 (FQ resistance)	CF	Ciprofloxacin or Levofloxacin
5	*Pseudomonas aeruginosa* (IMP, OXA−51)	*Pseudomonas aeruginosa*	Aminoglycosides, Beta-lactams	Cefepime	*Serratia marcescens*	blaSHV (Beta-lactamase)	CF	Meropenem or Piperacillin-Tazobactam
6	*Escherichia coli* (CTX-M−14, qnrB1)	*Escherichia coli*	Nitrofurantoin, TMP/SMX	Cefepime	*Enterococcus faecium*	VanB (VRE)	CF	Linezolid or Daptomycin
7	*Morganella morganii* (qnrD, blaCMY−2)	*Morganella morganii*	Fluoroquinolones, TMP/SMX	Cefepime	*Proteus mirabilis*	qnrS (FQ resistance)	CF	Meropenem or Ciprofloxacin
8	*Providencia stuartii* (blaNDM−1, qnrB4)	*Providencia stuartii*	Beta-lactams, Aminoglycosides	Cefepime	*Escherichia coli*	CTX-M−27 (ESBL)	CF	Colistin or Cefiderocol
9	*Escherichia coli* (CTX-M−15, qnrB1)	*Escherichia coli*	None detected	Trimethoprim-Sulfamethoxazole	None	CTX-M−15 (ESBL), qnrB1 (FQ resistance)	CF	Meropenem or Fosfomycin
10	*Enterococcus faecium* (VanA)	*Enterococcus faecium*	Tetracyclines	Amoxicillin	None	VanA (Vancomycin Resistance)	CF	Daptomycin or Linezolid
11	*Klebsiella pneumoniae* (OXA−48, CTX-M−15)	*Klebsiella pneumoniae*	Fluoroquinolones	Cephalexin	None	OXA−48 (Carbapenemase), CTX-M−15 (ESBL)	CF	Ceftazidime-Avibactam or Meropenem
12	*Pseudomonas aeruginosa* (VIM, OXA−51)	*Pseudomonas aeruginosa*	Beta-lactams (except Carbapenems)	Levofloxacin	None	VIM (Carbapenemase), OXA−51 (Beta-lactamase)	CF	Cefiderocol or Colistin
13	*Candida glabrata* (Azole Resistance) + *Escherichia coli* (CTX-M−27)	*Escherichia coli*	Trimethoprim-Sulfamethoxazole	Nitrofurantoin	None	Candida glabrata (Azole Resistance)	CF	Fluconazole or Echinocandins
14	*Klebsiella pneumoniae* (KPC−3, blaSHV−12)	*Klebsiella pneumoniae*	Fluoroquinolones	Amoxicillin-Clavulanate	None	KPC−3 (Carbapenemase), blaSHV−12 (Beta-lactamase)	CF	Ceftazidime-Avibactam or Meropenem
15	*Escherichia coli* (OXA−48, qnrS1)	*Escherichia coli*	Beta-lactams, Trimethoprim-Sulfamethoxazole	Doxycycline	None	OXA−48 (Carbapenemase), qnrS1 (FQ resistance)	CF	Meropenem or Fosfomycin
16	*Enterobacter cloacae* (qnrB1, blaCTX-M−15)	*Enterobacter cloacae*	Fluoroquinolones	Metronidazole	None	qnrB1 (FQ resistance), CTX-M−15 (ESBL)	CF	Meropenem or Fosfomycin

**Table 11:** Clinical Impact: C&S Missed Resistance, PCR-Detected Genes, and Appropriate Antibiotic Therapy (PCR Arm).

Case	Pathogen(s) Detected by PCR (with Resistance Marker)	Pathogen(s) Found in Culture	Phenotypic Resistance Detected in Culture	Antibiotic Prescribed Based on PCR (Guided Treatment)	Outcome of PCR-Guided Treatment	Antibiotic Would Have Been Prescribed Based on CS	Pathogen(s) Detected by PCR but Missed by Culture	Resistance Marker(s) Missed by Culture	Risk of Treatment Failure if Guided by CS?
1	*Escherichia coli* (qnrS, CTX-M−15)	*Escherichia coli*	Fluoroquinolones, TMP/SMX	Fosfomycin	FCl	Nitrofurantoin	None	qnrS (FQ resistance), CTX-M−15 (ESBL)	High
2	*Klebsiella pneumoniae* (CTX-M-Group1)	*Klebsiella pneumoniae*	Fluoroquinolones, Aminoglycosides	Amoxicillin and Clavulanate Potassium	FCl	Ciprofloxacin	None	CTX-M (Cephalosporin Resistance)	High
3	*Pseudomonas aeruginosa* (OXA−50, efflux-mediated FQ resistance)	*Pseudomonas aeruginosa*	Reduced FQ sensitivity	Levofloxacin	FCl	Ciprofloxacin	None	OXA−50 (Carbapenem Resistance), Efflux-mediated FQ resistance	High
4	*Enterococcus faecium* (VanA, ermB)	*Enterococcus faecium*	Aminoglycosides, Tetracyclines	Doxycycline	FCl	Nitrofurantoin	None	VanA (VRE), ermB (Macrolide resistance)	High
5	*Klebsiella pneumoniae* (OXA−48, CTX-M−15)	*Klebsiella pneumoniae*	Beta-lactams (except Carbapenems), Macrolides	Trimethoprim and Sulfamethoxazole	FCl	Amoxicillin/Clavulanate	None	OXA−48 (Carbapenem Resistance), CTX-M−15 (ESBL)	High
6	*Staphylococcus aureus* (mecA, ermC)	*Staphylococcus aureus*	Macrolides, Tetracyclines	Doxycycline	FCl	Cephalexin	None	mecA (MRSA), ermC (Macrolide resistance)	High
7	*Serratia marcescens* (IMP, blaSHV)	*Serratia marcescens*	Beta-lactams, Aminoglycosides	Levofloxacin	FCl	Ciprofloxacin	None	IMP (Carbapenemase), blaSHV (Beta-lactamase)	High
8	*Escherichia coli + Enterococcus faecalis* (VanA)	*Escherichia coli*	TMP/SMX, Aminoglycosides	Fosfomycin	FCl	Cephalexin	*Enterococcus faecalis*	VanA (VRE)	High
9	*Klebsiella pneumoniae + Pseudomonas aeruginosa* (OXA−50)	*Klebsiella pneumoniae*	Beta-lactams (except Carbapenems)	Ciprofloxacin	FCl	Amoxicillin/Clavulanate	*Pseudomonas aeruginosa*	OXA−50 (Carbapenem Resistance)	High
10	*Escherichia coli + Proteus mirabilis* (CTX-M−15)	*Escherichia coli*	TMP/SMX, Aminoglycosides	Nitrofurantoin	FCl	Ciprofloxacin	*Proteus mirabilis (missed due to E. coli overgrowth)*	CTX-M−15 (ESBL)	High
11	*Enterococcus faecalis + Klebsiella pneumoniae* (qnrB)	*Klebsiella pneumoniae*	Fluoroquinolones, Beta-lactams	Amoxicillin	FCl	Nitrofurantoin	*Enterococcus faecalis*	qnrB (FQ resistance)	High
12	*Pseudomonas aeruginosa + Escherichia coli* (CTX-M−27)	*Escherichia coli*	TMP/SMX, Cephalosporins	Ciprofloxacin	FCl	Cephalexin	*Pseudomonas aeruginosa*	CTX-M−27 (ESBL)	High
13	*Candida spp. + Escherichia coli*	*Escherichia coli*	TMP/SMX, Beta-lactams	Metronidazole + Fosfomycin	FCl	Cephalexin	*Candida spp.*	Antifungal Resistance	High
14	*Proteus mirabilis + Klebsiella pneumoniae* (OXA−48)	*Klebsiella pneumoniae*	Beta-lactams (except Carbapenems), Aminoglycosides	Amoxicillin and Clavulanate Potassium	FCl	Nitrofurantoin	*Proteus mirabilis*	OXA−48 (Carbapenem Resistance)	High
15	*Escherichia coli + Staphylococcus aureus* (ermC)	*Escherichia coli*	TMP/SMX, Beta-lactams	Trimethoprim and Sulfamethoxazole	FCl	Nitrofurantoin	*Staphylococcus aureus*	ermC (Macrolide Resistance)	High
16	*Klebsiella pneumoniae + Enterococcus faecalis* (VanB)	*Klebsiella pneumoniae*	Fluoroquinolones, TMP/SMX	Fosfomycin	FCl	Amoxicillin/Clavulanate	*Enterococcus faecalis*	VanB (VRE)	High
17	*Candida spp. + Enterococcus faecium* (VanA)	None (Culture Negative)	None	Metronidazole + Nitrofurantoin	FCl	Cephalexin	*Candida spp., Enterococcus faecium*	VanA (VRE), Antifungal Resistance	High

## Data Availability

Due to the sensitive nature of the clinical data and patient confidentiality requirements, the datasets used and/or analyzed during the current study are not publicly available. However, they are available from the corresponding author on reasonable request, provided that the request complies with relevant ethical guidelines and data protection regulations.

## Supplementary Materials

The following supporting information can be downloaded at: https://www.mdpi.com/article/10.3390/microorganisms13040949/s1, The supplementary materials provided detail the methodologies used for both polymerase chain reaction (PCR) and culture and sensitivity (C&S) analyses conducted in the study. For the PCR methodology, the supplement outlines the specific primers employed for pathogen detection, the composition of the reaction mix, including reagents such as dNTPs, primers, Taq polymerase, and the DNA template, as well as the thermal cycling conditions used during amplification. It also describes the method for visualizing amplified products via agarose gel electrophoresis stained with ethidium bromide. For the C&S methodology, the document includes the types of culture media used for bacterial isolation, such as Blood Agar and MacConkey Agar, and specifies incubation conditions. It also provides details of the antibiotic sensitivity testing protocol, including the disk diffusion method and the criteria used to interpret zones of inhibition in line with clinical guidelines. These materials collectively support the reproducibility and transparency of the microbial identification and antimicrobial resistance testing employed in the study.
