# Supplementary material for: The Clinical Validity and Utility of PCR Compared to Conventional Culture and Sensitivity Testing for the Management of Complicated Urinary Tract Infections in Adults: A Secondary (Ad Hoc) Analysis of Pathogen Detection, Resistance Profiles, and Impact on Clinical Outcomes
Source: Microorganisms. 2025 Apr 20;13(4):949. doi: 10.3390/microorganisms13040949 (PMC12029264; doi:10.3390/microorganisms13040949)
Supplement: Supplementary file 1 [file microorganisms-13-00949-s001.zip › microorganisms-3572522-supplementary.pdf]

Status Active PolicyStat ID 15968603

Origination Date	09/2014	Owner	Diana Wilkerson: Dept Director
Last Approved	06/2024	Policy Area	Laboratory Microbiology
Effective	06/2024		
Last Revised	06/2024		
Next Review	06/2026		

Urine Culture

COPY

PRINCIPLE:

Cultures from the Urinary tract can be submitted for identification of aerobic bacteria.

NOTE: Suprapubic collections may be received in the lab with the needle attached so extreme caution must be exercised.

SPECIMENS:

CLEAN CATCH: The patient is given a sterile clean catch midstream collection kit that contains towelettes. The patient is given instructions on collection; The patient is to use the first towelette to cleanse the genital area thoroughly. The patient is instructed to begin urination into the commode. After a brief period of urination the patient is instructed to stop urination and use the second towelette to again thoroughly cleanse the genital area. The patient should begin urination into the sterile container. When partially full the container may be set aside and the patient should finish urinating into the commode. The last towelette is to clean the genital area and/or hands prior to placing the lid on the collection container.

CATHERIZATION:

This is an invasive procedure and is performed by qualified nursing personnel (not laboratory staff). Indwelling catheters are clamped off for a short period of time by nursing personnel. The aspiration port is thoroughly cleaned prior to sampling. A minimum of a few mL is necessary for a culture. The clamp is released.

SUPRAPUBIC ASPIRATION:

This invasive procedure will be performed by a Physician. The specimen is labeled, orders are written and the sample and orders are transported to the lab as soon as possible.

SUPPLIES/REAGENTS:

Bi plate (if a bi plate is unavailable,use Blood agar and MacConkey agar.) 1 uL Sterile loops (if 10 uL loop is used, multiply count by an factor of 100. Be sure to indicated on the label that the 10 uL was used to streak the BAP plate).

1. The specimen is received in the lab and labels are generated using the computer.

2. Label the bi plate with the correct patient label.

3. For the BAP side, a sterile 1ul loop is used to streak down the middle and then the loop is used to streak the plate in colony count manner (at a 90 degree angle to original streak).

4. The MacConkey side is streaked for isolation.

5. Place all plates in the incubator.

6. Plates are examined daily for pathogens for up to 2 days.

Refer to the ASM Handbook for guidance.

RESULTS:

COPY

NEGATIVE RESULTS WILL BE REPORTED USING THE FOLLOWING FORMAT:

1. No Growth at 24 hours (preliminary)

2. No Growth at 48 hours (final)

Cultures showing growth will follow these guidelines:

Catheter collected, suprapubic , kidney and nephrostomy specimens , as well as pediatric patient specimens have a more stringent algorithm when working up cultures. Refer to ASM Handbook for guidance. (Generally, most uropathogenic organisms will be worked up.)

For Voided urines, the general rule is if there are 3 or more organisms , the urine is reported as contaminated. This is up to the technicians judgement based on factors such as amount of each organism present, the results of the urinalysis , the patient's information / diagnosis, etc.

For contaminated urines , the Cerner(LIS) codes used for reporting are 3DC1 (3 or more organisms present, possible contamination.) and 3DC2 (Suggest recollection if patient symptomatic.)

For pathogenic organisms worked up , use the following format:

(Colony count) (organism)

If applicable, MIC breakpoint report is included.

There are many variables which can affect the reporting of urine cultures. Techs must use their good judgement. Refer to ASM Handbook for guidance.

REFERENCES:

1. Koneman, E. W., Color Atlas and Textbook of Diagnostic Microbiology, J. B. Lippincott Co., Philadelphia, PA. p.37-38.

Approval Signatures

Step Description	Approver	Date
Director Review	Harry Latham MD: Pathologist	06/2024
Manager Review	Zack Alexander: Lab Operations Lead	06/2024
Dept Director	Diana Wilkerson: Dept Director	06/2024

COPY

Crisp Regional Hospital, Inc.
Harry S. Latham MD, Lab Director CLIA # 11D0646027
902 North 7th Street * Cordele, GA 31015

**Name:**	[redacted]		Admit Date:	
MRN / FIN:	[redacted]	[redacted]	Discharge Date:	
DOB / Age:	[redacted]	64 years	Location:	CRRH RAD
Sex / Birth Sex:	Female	Female	Ordering:	White,William K MD

Urinalysis

Legend: @=Abnormal !=Critical L=Low H=High c=Corrected f=Comment O=Order Comment i=Interp Data *=Performing Lab

UA Macroscopic

Collected Date	6/20/2023		
Collected Time	10:44 EDT		
Procedure		Units	Reference Range
Urine Srce	Clean Catch		
UA Color	Yellow		
UA Appear	Clear		
UA pH	6.0		
UA Spec Grav	1.020		[1.005-1.030]
UA Glucose	3+		
UA Bili	NEGATIVE		
UA Ketones	NEGATIVE		
UA Blood	1+		
UA Protein	3+		
UA Urobilinogen	0.2		
UA Nitrite	Negative		
UA Leuk Est	NEGATIVE		[>=0]
UA Micro Ind?	Indicated		
UA Culture Indicated?	Indicated		

UA Microscopic

Collected Date	6/20/2023		
Collected Time	10:44 EDT		
Procedure		Units	Reference Range
UA WBC	Moderate		
UA RBC	Rare		
UA Bacteria	Moderate		
UA Squam Epithelial	Rare		
UA Yeast	Present		
UA Fine Gran	Present		

Crisp Regional Hospital, Inc.
Harry S. Latham MD, Lab Director CLIA # 11D0646027
902 North 7th Street * Cordele, GA 31015

**Name:**	[redacted]		Admit Date:	
MRN / FIN:	[redacted]	[redacted]	Discharge Date:	
DOB / Age:	[redacted]	64 years	Location:	CRRH RAD
Sex / Birth Sex:	Female	Female	Ordering:	White,William K MD

Microbiology

Legend: c=Corrected f=Comment *=Performing Lab S=Susceptible I=Intermediate R=Resistant N/A=Not Applicable

Procedure:	Urine Culture		
Source:	Urine	Accession:	13-23-171-0193
Collected Date/Time:	6/20/2023 10:44 EDT	Body Site:	
Start Date/Time:	6/20/2023 14:07 EDT	Free Text Source:	

FINAL REPORTS
Final Report
Verified Date/Time: 6/22/2023 08:59 EDT
>100,000 cfu/mL Escherichia coli

PRELIMINARY REPORTS
Preliminary Report
Verified Date/Time: 6/21/2023 09:40 EDT
>100,000 cfu/mL Gram Negative Bacilli
Identification and sensitivity to follow

SUSCEPTIBILITY RESULTS

	Escherichia coli	
Antibiotic	MIC Dilutn	MIC U Int
Amikacin	<=16	Susceptible
Ampicillin	>16	Resistant
Ampicillin/Sulbactam	>16/8	Resistant
Aztreonam	<=4	Susceptible
Cefazolin	<=2	Susceptible
Cefepime	<=2	Susceptible
Cefoxitin	<=8	Susceptible
Ceftazidime	<=1	Susceptible
Ceftriaxone	<=1	Susceptible
Ciprofloxacin	<=1	Susceptible
Ertapenem	<=0.5	Susceptible
Gentamicin	<=4	Susceptible
Imipenem	<=1	Susceptible
Levofloxacin	<=2	Susceptible
Meropenem	<=1	Susceptible
Nitrofurantoin	<=32	Susceptible
Tigecycline	<=2	Susceptible
Tobramycin	<=4	Susceptible
Trimethoprim/Sulfamethoxazole	<=2/38	Susceptible

Bacteria
Citrobacter freundii/braakii
Gardnerella vaginalis
Staphylococcus (coagulase negative: epidermidis, haemolyticus, lugdunensis, saprophyticus)
Acinetobacter baumannii
Klebsiella pneumoniae/oxytoca
Staphylococcus aureus
Streptoccoccus agalactia (group B)
Escherichia coli
Serratia marcescens
Staphylococcus saprophyticus
Proteus mirabilis, vulgaris
Citrobacter koseri
Pseudomonas aeruginosa
Streptococcus pyogenes
Enterococcus faecium, faecalis

Fungi
Candida albicans, glabrata, parapsilosis, tropicalis

STI
Mycoplasma genitalium
Ureaplasma urealyticum
Neisseria gonorrhoeae
Trichomonas vaginalis
Chlamydia trachomatis

Resistance Genes
PER-1/VEB-1/GES-1 Groups (ESBL)
VanA, VanB (Vancomycin)
IMP, NDM, VIM Groups (Carbapenem)
Class A ß-lactamase; CTX-M-Group1
Class D oxacillinase OXA--51
qnrA1, A2
tetB, tetM
ACT, MIR, FOX, ACC Groups (Beta Lactams)
Class B metallo-ß-lactamase; blaNDM
dfr (A1, A5), sul (1,2) probes (Sulfamethoxazole and trimethoprim)
qnrS
MRSA* Mec-A
Class A ß-lactamase; blaKPC
Class D oxacillinase OXA-48
ermB, C; mefA
qnrB

1. **Purpose:** This procedure defines the responsibilities and describes the process used for extracting and purifying Total Nucleic acid from clean catch urine samples using the MagMAX™ Viral/Pathogen Ultra Total Nucleic Acid Kit on KingFisher Duo Prime and then running on the Thermo Fisher QuantStudio 7 Flex. TaqMan® Array Card is an efficient, easy-to-use system for the characterization of key microbial targets. This panel includes TaqMan® assays that have been optimized for the detection of 28 organisms, which include bacterial, fungal and parasitic targets along with antibiotic resistance to 16 drugs. Control assays i.e. TaqMan® Universal Extraction Control Organism, and TaqMan® Amplification controls are included to track the overall quality of the entire process.
2. **Materials:**
   - 2.1. Components of the MagMax™ Viral/Pathogen Ultra Nucleic Acid Isolation Kit (Cat# A42356)- stored at 15℃ to 25℃
     - 2.1.1. Binding Solution 53 mL
     - 2.1.2. Wash Buffer 100 mL
     - 2.1.3. Elution Solution 10 mL
     - 2.1.4. Proteinase K 1 mLTotal Nucleic Acid Binding Beads 2 mL
     - 2.1.5. Enzyme mix 5 mL (stored at -15℃ to -25℃)
   - 2.2. TaqPath 1-Step RT-qPCR Master Mix -15℃ to -25℃
   - 2.3. KingFisher™ deep-well 96 plate
   - 2.4. Duo Prime 12-Tip comb for use in deep well 96 plate
   - 2.5. KingFisher™ Duo Elution strip and cap
   - 2.6. UTI TaqMan card (2℃ to 8℃)
3. **Specimens:**
   - 3.1. Specimens should be collected and transported as per the established SOP.
   - 3.2. Specimens can be stored refrigerated (2-6°C) for up to 48 hours. If testing is prolonged, the sample can be stored frozen for up to one month.
   - 3.3. Any remaining stock nucleic acid will be stored at -15°C to -25°C for a minimum of one month.
4. **Quality Control:**
   - 4.1. TaqMan® Universal Extraction Control Organism serves as a process control for the DNA extraction and purification process and is processed throughout the entire PCR workflow. TaqMan® Universal Extraction Control Organism is Bacillus atrophaeus, a gram-positive bacteria whose structural characteristics make it an ideal control for sample extraction and purification. (stored at -15℃ to -25℃)
   - 4.2. TaqMan® Urinary Tract Microbiota Amplification Control contains a linearized multi-target plasmid with target sequences for each available urinary tract microbiota profiling assay. It can be included in profiling experiments as a positive control and for troubleshooting. It will be included on the first UTI card run weekly as a stand-alone

sample at real-time PCR and carried through the remainder of the workflow. (stored at -15℃ to -25℃)

4.3. Negative Control is well that remains empty throughout the DNA extraction procedure, to which Nuclease-Free Water is added directly to the elution plate following the extraction and will be used in the Taq card to check for any possible carryover.

5. Setting up the 96 Deep Well Sample Plate

5.1. Take the Enzyme mix and *Bacillus atrophaeus* out of the freezer.

5.2. Invert the Patient's Urine Sample 10X or swirl for 10 sec.

5.3. Label microfuge tube for corresponding urines.

5.4. Aliquot 1000 uL of urine to the appropriate microfuge tube and spin for 5 mins at 9000 RPM.

While waiting for urines to spin down, Steps 5.5-5.7 can be completed.

5.5. Add 1000 uL of Wash Buffer in Row C of 96 Deep Well Sample Plate for every sample that will be tested.

5.6. Add 1000 uL of 80% Ethanol to Row E of 96 Deep Well Sample Plate for every sample that will be tested.

5.7. Add 500 uL of 80% Ethanol to Row G of 96 Deep Well Sample Plate for every sample that will be tested.

5.8. Decant Urine by pipetting ~900 uL from the microfuge tube down to the 0.1 uL mark so as to not disturb the pellet and discard along with the pipette tip.

5.9. Add 400 uL PBS (Phosphate Buffered Saline) to each microfuge tube.

5.10. Vortex each microfuge tube with urine/PBS sample for 10-20 seconds to resuspend the pellet.

5.11. Add the following to Row A of 96 Deep Well Sample Plate for every sample that will be tested:

5.11.1. 50 uL Enzyme Mix - Pipette slowly, highly viscous.

5.11.2. 10 uL B. atrophaeus control - change tip between each well.

5.11.3. 400 uL of urine suspended in PBS - change tips between each well/sample. Discard tubes after being transferred to wells.

5.12. Put a new Tip Comb in 96 Deep Well Sample Plate Row H.

Plate Row	Reagent	Volume per Well
**A**	**Sample**	**~ 500 uL**
B	Empty	
**C**	**Wash Buffer**	**1,000 uL**
D	Empty	
**E**	**80% Ethanol**	**1,000 uL**
F	Empty	
**G**	**80% Ethanol**	**500 uL**
H	12 Tip Comb	

5.13. Load 96 Deep Well Sample Plate onto KingFisher with the A1 cell in the corresponding position on KingFisher.

5.14. Add 100 uL of Pathogen Elution Solution to the Elution Strip for every sample that will be tested.

Plate Row	Reagent	Volume per Well
**A**	**Elution Solution**	**100 uL**

5.15. Select MVP_Ultra_Duo and Press Start □

5.16. Follow the prompts on the screen and then press the ✔.

While waiting for the 1st part of the KingFisher run, steps 5.17 - 5.20 can be done in preparation.

5.17. Put Enzyme Mix and *B. atrophaeus* back into the freezer.

5.18. In a 50mL conical tube, add 530 uL of Binding Solution per sample tested plus two extra.

5.19. In the same 50mL conical tube, add 20 uL of binding Beads per sample tested plus two extra. Vortex the binding beads before adding them to the solution.

5.20. Invert or swirl the tube with Binding Bead Solution to mix, DO NOT VORTEX!

5.21. After ~20 minutes the KingFisher will ask for Binding Bead Solution and Proteinase K.

5.22. Remove the 96 Deep Well Sample Plate from the KingFisher.
5.23. Add 10 uL of Proteinase K to Row A of 96 Deep Well Sample Plate for every sample that will be tested.
  5.23.1. Change the pipette tip if the tip comes in contact with the inside of the sample well.
5.24. Add 550 uL of Binding Bead Solution (invert or swirl before use) to Row A of 96 Deep Well Sample Plate for every sample that will be tested.
  5.24.1. Change the pipette tip if the tip comes in contact with the inside of the sample well.
5.25. Load the 96 Deep Well Sample Plate back onto the KingFisher Duo and press the ✔.
5.26. Take the UTI TaqMan card out of the refrigerator and Master Mix out of the freezer to bring it to room temperature.

6. Preparing TaqMan card for analysis on QuantStudio 7 Flex:

6.1. Label microfuge tubes to correspond with the samples being tested.
6.2. Add the following to each microfuge tube:
  6.2.1. 32 uL of UltraPure DI H2O
  6.2.2. 28 uL of 1-Step RT-qPCR Master Mix (DO NOT VORTEX!)
6.3. Label one microtube **NTC** and add the following:
  6.3.1. **82 uL** of UltraPure **DI H2O**
  6.3.2. **28 uL** of 1-Step RT-qPCR **Master Mix** (DO NOT VORTEX!)
6.4. After the extraction on the KingFisher is complete ~30 minutes, discard the 96 Deep Well Sample Plate in BioHazard.
6.5. Take the elution strip off the KingFisher and transfer 50 uL of elution into the corresponding microfuge tube.
  6.5.1. Discard the pipette tip after each sample.
  6.5.2. Discard the elution strip after all samples have been transferred.
6.6. Mix sample 5-6 times with pipette fill port on UTI TaqMan card with 100 uL of the corresponding sample from microfuge tube and discard tube.
  6.6.1. Dispense samples slowly and steadily so as not to introduce bubbles.
6.7. Fill the 8th port on the UTI TaqMan card with 100 uL UltraPure DI H2O - to be used as an NTC (No Template Control).
6.8. Centrifuge TaqMan card at 1200 rpm for 1 minute.
6.9. Repeat step 6.7.
  6.9.1. If the card appears to have any bubbles, you can repeat step 6.7 again, but DO NOT centrifuge the card more than 3 times.
  6.9.2. Seal the card using the TaqMan Array Card Sealer by only pushing forward.
    6.9.2.1. DO NOT pull the handle back towards you!
  6.9.3. Cut off the foil tip near the edge of the plastic where the open ports were.

7. Loading Card and Running Experiment on QuantStudio 7 Flex:

7.1. Open Door on Flex (can be done in two different ways).
 7.1.1. Press Red Up Arrow on the screen of Flex.
 7.1.2. From QuantStudio software: Select Console and choose "Open Door" on the Top Task Bar.
7.2. Load the Sealed TaqMan card onto the arm with A1 in the top left corner and the barcode facing you.
7.3. Shut the door on Flex using either of the two ways mentioned above.
7.4. Select "New Experiment" from the top left drop-down menu → From Template
 7.4.1. Choose Office Template Folder
 7.4.2. Select Custom UTM Template and open.

Experiment Type: Array Card, Experiment: Comparative Ct, Run Type: Fast, Import the plate file for card, change the run method settings to the following...				
**Step**	**Stage**	**Cycles**	**Temp.**	**Time**
UNG incubation	1	1	25℃	2 minutes
Reverse Transcription	2	1	50℃	15 minutes
Polymerase Activation	3	1	95℃	2 minutes
Amplification	4	40	95℃	3 seconds
			60℃	30 seconds

7.5. On Q12 Software Experiment Menu, choose Define under the setup option.
 7.5.1. Change Sample Names to correspond with the samples being tested on the card.
7.6. Go to Run.
 7.6.1. The Green Drop Down Menu "Start Run"
 7.6.2. Choose Console.
 7.6.3. Choose the folder to save the run to and assign the name run following lab standards.
7.7. Go back to the Green Drop Down Menu "Start Run"
 7.7.1. Choose Console and your run will start.
7.8. ~1 hour 30 minutes, the experiment will be over. Unload the used card following steps 7.1 - 7.3 except removing the card and discarding it in the BioHazard waste bin.

8. **References:**
   8.1. Urinary Tract Microbiota Profiling Experiments: APPLICATION GUIDE TaqMan® Fast Virus 1-Step Master Mix.
   8.2. MagMAX™ Viral/Pathogen Ultra Total Nucleic Acid Extraction - KF Duo Prime Protocol EA.

Version	Date	Reason for Revision	Person Making Revision
1.0	10/1/2021	Original Release	NA

1. **Purpose:** The purpose of this procedure is to standardize how to interpret positive and negative results for all assays run using the QuantStudio 7 Flex software.
2. **Materials:**
   2.1. QuantStudio 7 Software
   2.2. TrueMed LIS
3. **Procedure:**
   3.1. The following Ct ranges are used for reporting all pathogens.
      3.1.1. **Critically High:** 10.000 - 21.000
      3.1.2. **High:** 22.000 - 26.000
      3.1.3. **Medium:** 27.000 - 28.000
      3.1.4. **Low:** 29.000 - 32.000
      3.1.5. **Negative:** 40.000 - 33.000; 09.000 - 1.000
   3.2. The following Ct ranges are used for reporting all resistance genes.
      3.2.1. **Positive:** 10.000 - 32.000
      3.2.2. **Negative:** 33.000 - 40.000; 1.000 - 9.000
   3.3. In order to produce a valid result for the report, you will need to take into account the C_t value generated, the amplification plot graph, and the multicomponent plot graph if the amplification graph is unclear.
      3.3.1. **Amplification:** Record pathogen/resistance gene as positive if it contains C_t value and amplification (example below shows E.Coli with a C_t value of 25.2 and amplification)

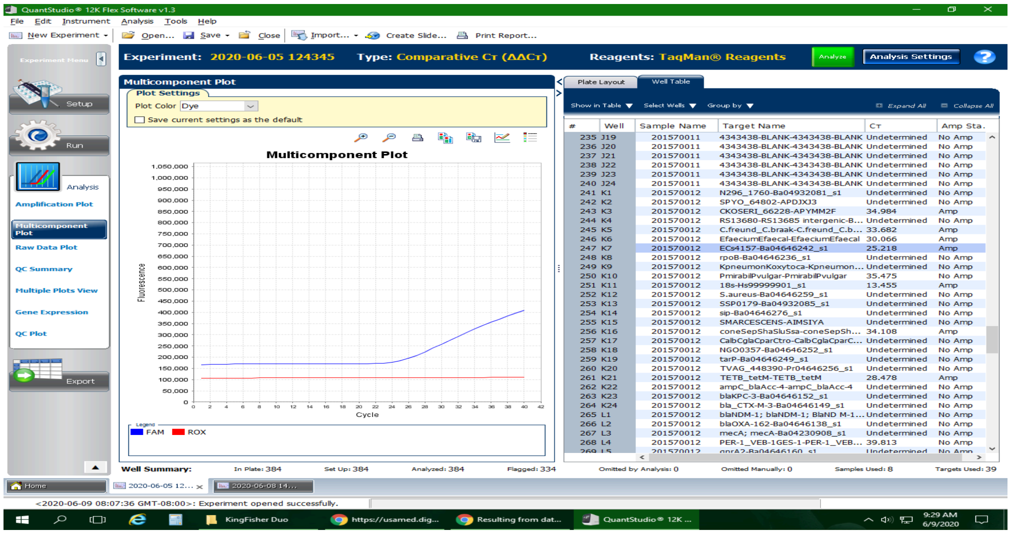

3.3.2. Record the pathogen/resistance gene as negative if the Ct value is undetermined (whether amplification is present or absent).

3.3.3. Record pathogen/resistance gene as negative if Ct value is present and there is no amplification (example below shows Proteus mirabilis, vulgaris Ct value of 35.4 and no amplification).

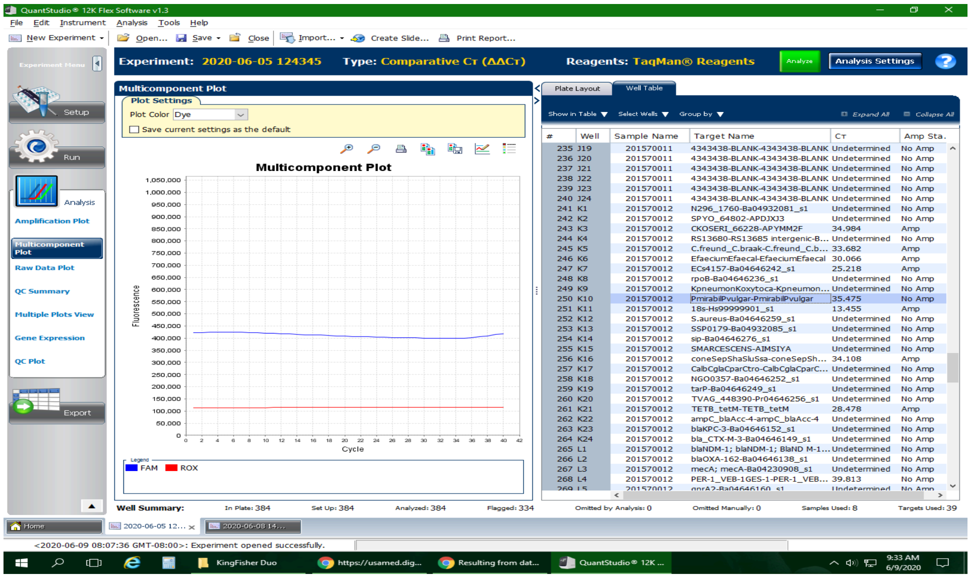

3.3.4. Check the amplification and multicomponent plot graphs for each target containing a Ct value and the amplification status is inconclusive.

3.3.4.1. If the graph shows evidence of amplification, record the pathogen/resistance gene as positive/negative based on the Ct value. If you cannot conclude that there is amplification, record the pathogen/resistance gene as negative.

3.3.5. To rule out any false positives, if the Ct value for a given pathogen/resistance gene is over 30 and the curve on the amplification plot starts after cycle 32, report the pathogen/resistance gene as negative.

3.3.5.1. If the Ct value for a pathogen/resistance gene is over 30 and the curve on the multicomponent plot is amplified after cycle 30, report the pathogen/resistance gene negative.

3.3.6. **Amp Score:** If there is an Amp Score of 1.2 or greater, amplification and a curve seen on the amplification plot or multicomponent plot, then record the pathogen/resistance gene as positive. If the Amp Score is less than 1.2, amplification and a curve seen on the amplification plot or multicomponent plot

record the pathogen/resistance gene as positive. If the Amp Score is less than 1.2, the amplification and **NO** curve seen on the amplification plot or multicomponent plot record the pathogen/resistance gene as negative.

3.3.7. **CQ Conf:** The CQ conf score is an additional confirmatory quantitative measure that can determine the presence or absence of a pathogen/resistance gene. If criteria for C_t, and Amp Score are met, the CQ conf can be used for confirmation. If the CQ conf is 0.90 or greater, record the pathogen/resistance gene as positive.

4. Quality Controls:

4.1. The *B. atrophaeus* control must have a Ct value within our reportable range and amplification before you can report out that sample.

Version	Date	Reason for Revision	Person Making Revision
1.0	10/01/2021	Original Release	NA

UTI PCR Report

Final Report

Patient Information	Provider Information	Specimen Information
**Patient Name:** UTI 5, UTI 5	**Ordering Physician:** test	**Accession ID:** 250590120
**Date of Birth:** 8/7/1986	**Lab Director:** Ying Liu, M.D.	**Specimen Type:** Urine
**Age:** 38 Years	**CLIA#:** 02D0640642	**Collection Date:** 02/28/2025
**Sex:** Male		**Result Date:** 2/28/2025 1:05:25 PM

Result Summary

Organism(s) Tested - Detected:

Organism Detected	Est. Microbial Load*	Total % Pathogen Load	Potential Therapeutic Agents
Klebsiella pneumoniae/oxytoca	32.768*10^5 copies/mL	100.0000000%	- Linezolid<br>- Nitrofurantoin<br>- Cefiderocol<br>- Aztreonam<br>- Moxifloxacin<br>- Ofloxacin<br>- Piperacillin/Tazobactam<br>- Amoxicillin/Clavulanate (Augmentin) (PO)<br>- Amoxicillin/Clavulanate (Augmentin)<br>- Piperacillin/Tazobactam (IV)<br>- Ticarcillin/Clavulanate<br>- Cefazolin (Ancef)<br>- Cefazolin (Ancef) - 1st Gen<br>- Cefepime<br>- Gentamicin<br>- Amikacin<br>- Plazomicin<br>- Ceftriaxone - 3rd Gen<br>- Ceftriaxone<br>- Cefepime (IV) - 4th Gen<br>- Levofloxacin po/IV<br>- Levofloxacin<br>- Ciprofloxacin po/IV<br>- Ciprofloxacin<br>- Fosfomycin<br>- Colistin

Antibiotic Resistance Detected:

Resistance Gene Detected	Resistant Against
Class A ß-lactamase; blaKPC	Carbapenems
dfr (A1, A5), sul (1,2) probes (Sulfamethoxazole and trimethoprim)	Sulfonamides

UTI PCR Report

UTI 5, UTI 5 8/7/1986 Accession ID: 250590120

ALASKA UROLOGY

Medications:

	Klebsiella pneumoniae/oxytoca
Amikacin	S
Amikacin IV	-
Amoxicillin	-
Amoxicillin PO	-
Amoxicillin/Clavulanate (Augmentin)	S
Amoxicillin/Clavulanate (Augmentin) (PO)	S
Amphotericin B	-
Ampicillin	-
Ampicillin PO	-
Ampicillin/Sulbactam	-
Azithromycin	-
Azithromycin PO	-
Aztreonam	S
Cefazolin (Ancef)	S
Cefazolin (Ancef) - 1st Gen	S
Cefazolin (Ancef)	-
Cefdinir	-
Cefdinir PO - 3rd Gen	-
Cefepime	S
Cefepime (IV) - 4th Gen	S
Cefiderocol	S
Cefixime	-
Cefixime PO - 3rd Gen	-
Cefpodoxime	-
Cefpodoxime - 3rd Gen	-
Cefprozil	-
Ceftaroline fosamil (Teflaro)	-
Ceftazidime	-
Ceftazidime - 3rd Gen	-
Ceftriaxone	S
Ceftriaxone - 3rd Gen	S
Cefuroxime	-
Cephalexin (Keflex)	-
Cephalexin (Keflex) - 1st Gen	-
Cephalexin (Keflex)	-
Chloramphenicol	-
Ciprofloxacin	S
Ciprofloxacin po/IV	S
Clarithromycin	-
Clindamycin	-
Clindamycin PO	-

UTI PCR Report

UTI 5, UTI 5   8/7/1986   Accession ID: 250590120

	Klebsiella pneumoniae/oxytoca
Clotrimazole	-
Colistin	S
Dicloxacillin	-
Dicloxacillin PO	-
Doripenem	R
Doxycycline	-
Doxycycline po/IV	-
Ertapenem	R
Ertapenem IV	R
Erythromycin	-
Erythromycin PO	-
Fluconazole	-
Fluconazole po/IV	-
Flucytosine	-
Fosfomycin	S
Gemifloxacin	-
Gentamicin	S
Imipenem/Cilastatin	R
Imipenem/Cilastatin IV	R
Isavuconazole	-
Itraconazole	-
Ketoconazole	-
Levofloxacin	S
Levofloxacin po/IV	S
Linezolid	S
Linezolid PO	-
Meropenem	R
Meropenem IV	R
Metronidazole	-
Metronidazole (IV/po)	-
Miconazole	-
Minocycline	-
Moxifloxacin	S
Nafcillin	-
Nistatin	-
Nitrofurantoin	S
Nitrofurantoin (PO)	-
Ofloxacin	S
Oxacillin	-
Penicillin G	-
Piperacillin/Tazobactam	S
Piperacillin/Tazobactam (IV)	S
Plazomicin	S

UTI PCR Report

UTI 5, UTI 5 8/7/1986 Accession ID: 250590120

	Klebsiella pneumoniae/oxytoca
Posaconazole	-
Pristinamycin	-
Terbinafine	-
Ticarcillin/Clavulanate	S
TMP-SMX (Bactrim)	R
TMP-SMX (Bactrim) PO	R
TMP-SMX (Bactrim)	-
Tobramycin	-
Tolnaftate	-
Vancomycin	-
Voriconazole	-

Antibiotic Table Legend:
-: Not first choice [gray dash]
S: Sensitive [blue]
S^1: Sensitive (very effective) [green]
S^2: Sensitive (no other alternative) [orange]
S^3: Sensitive (do not use for pneumonia) [pink]
R: Treatment option that may be affected by the detected resistance marker. [Red]

Urinary Tract Infectious Disease Pathogens

Organism	Results	Est. Microbial Load*	Normal Reference Range
Candida albicans, glabrata, parapsilosis, tropicalis	Not Detected	Negative	Not Detected
Chlamydia trachomatis	Not Detected	Negative	Not Detected
Citrobacter freundii/braakii	Not Detected	Negative	Not Detected
Citrobacter koseri	Not Detected	Negative	Not Detected
Enterococcus faecium, faecalis	Not Detected	Negative	Not Detected
Escherichia coli	Not Detected	Negative	Not Detected
Gardnerella vaginalis	Not Detected	Negative	Not Detected
Klebsiella pneumoniae/oxytoca	Detected	High	Not Detected
Mycoplasma genitalium	Not Detected	Negative	Not Detected
Neisseria gonorrhoeae	Not Detected	Negative	Not Detected
Proteus mirabilis, vulgaris	Not Detected	Negative	Not Detected
Pseudomonas aeruginosa	Not Detected	Negative	Not Detected
Serratia marcescens	Not Detected	Negative	Not Detected
Staphylococcus (coagulase negative: epidermidis, haemolyticus, lugdunensis, saprophyticus)	Not Detected	Negative	Not Detected
Staphylococcus aureus	Not Detected	Negative	Not Detected

UTI PCR Report

UTI 5, UTI 5 8/7/1986 Accession ID: 250590120

Organism	Results	Est. Microbial Load*	Normal Reference Range
Staphylococcus saprophyticus	Not Detected	Negative	Not Detected
Streptococcus agalactia (group B)	Not Detected	Negative	Not Detected
Streptococcus pyogenes	Not Detected	Negative	Not Detected
Trichomonas vaginalis	Not Detected	Negative	Not Detected
Ureaplasma urealyticum	Not Detected	Negative	Not Detected
Acinetobacter baumannii	Not Detected	Negative	Not Detected

Antibiotic Resistance

Resistance Gene(s)	Antibiotic Class	Results	Est. Microbial Load*	Normal Reference Range
Class A ß-lactamase; blaKPC	N/A	Detected	N/A	Not Detected
Class A ß-lactamase; CTX-M-Group1	N/A	Not Detected	N/A	Not Detected
Class B metallo-ß-lactamase; blaNDM	N/A	Not Detected	N/A	Not Detected
Class D oxacillinase OXA-48	N/A	Not Detected	N/A	Not Detected
Class D oxacillinase OXA--51	N/A	Not Detected	N/A	Not Detected
dfr (A1, A5), sul (1,2) probes (Sulfamethoxazole and trimethoprim)	N/A	Detected	N/A	Not Detected
ermB, C; mefA	N/A	Not Detected	N/A	Not Detected
IMP, NDM, VIM Groups (Carbapenem)	N/A	Not Detected	N/A	Not Detected
MRSA* Mec-A gene	N/A	Not Detected	N/A	Not Detected
PER-1/VEB-1/GES-1 Groups (ESBL)	N/A	Not Detected	N/A	Not Detected
qnrA1, A2, B2	N/A	Not Detected	N/A	Not Detected
qnrB	N/A	Not Detected	N/A	Not Detected
qnrS	N/A	Not Detected	N/A	Not Detected
tetB,tetM	N/A	Not Detected	N/A	Not Detected
VanA, VanB (Vancomycin)	N/A	Not Detected	N/A	Not Detected
ACT, MIR, FOX, ACC Groups (Beta Lactams)	N/A	Not Detected	N/A	Not Detected

Analyzed By: Andrea Fragapane **Date:** 2/28/2025

Disclaimer: This test was developed and its performance characteristics determined by Alaska Urology @ Providence Laboratories.It has not been cleared or approved by the US Food and Drug Administration(FDA).FDA does not require this test to go through premarket FDA review.This test is used for clinical purposes. It should not be regarded as investigational or for research.This laboratory is certified under the Clinical Laboratory Improvement Amendments(CLIA) as qualified to perform high complexity clinical laboratory testing.
